# Targeting Oxidative Phosphorylation-Proteasome Activity in Extracellular Detached Cells Promotes Anoikis and Inhibits Metastasis

**DOI:** 10.3390/life12010042

**Published:** 2021-12-28

**Authors:** Funmilayo O. Adeshakin, Adeleye O. Adeshakin, Zhao Liu, Jian Cheng, Pengchao Zhang, Dehong Yan, Guizhong Zhang, Xiaochun Wan

**Affiliations:** 1 Guangdong Immune Cell Therapy Engineering and Technology Research Center, Center for Protein and Cell-Based Drugs, Institute of Biomedicine and Biotechnology, Shenzhen Institute of Advanced Technology, Chinese Academy of Sciences, Shenzhen 518055, China; funmilayo@siat.ac.cn (F.O.A.); adeleye.adeshakin@stjude.org (A.O.A.); zhao.liu@siat.ac.cn (Z.L.); jian.cheng@siat.ac.cn (J.C.); zhangpc@siat.ac.cn (P.Z.); dh.yan@siat.ac.cn (D.Y.); 2 University of Chinese Academy of Sciences, Beijing 100864, China

**Keywords:** anoikis, metastasis, oxidative phosphorylation, proteasome, AMPK

## Abstract

Metastasis arises owing to tumor cells’ capacity to evade pro-apoptotic signals. Anoikis—the apoptosis of detached cells (from the extracellular matrix (ECM)) is often circumvented by metastatic cells as a result of biochemical and molecular transformations. These facilitate cells’ ability to survive, invade and reattach to secondary sites. Here, we identified deregulated glucose metabolism, oxidative phosphorylation, and proteasome in anchorage-independent cells compared to adherent cells. Metformin an anti-diabetic drug that reduces blood glucose (also known to inhibit mitochondrial Complex I), and proteasome inhibitors were employed to target these changes. Metformin or proteasome inhibitors alone increased misfolded protein accumulation, sensitized tumor cells to anoikis, and impaired pulmonary metastasis in the B16F10 melanoma model. Mechanistically, metformin reduced cellular ATP production, activated AMPK to foster pro-apoptotic unfolded protein response (UPR) through enhanced expression of CHOP in ECM detached cells. Furthermore, AMPK inhibition reduced misfolded protein accumulation, thus highlight relevance of AMPK activation in facilitating metformin-induced stress and UPR cell death. Our findings provide insights into the molecular biology of anoikis resistance and identified metformin and proteasome inhibitors as potential therapeutic options for tumor metastasis.

## 1. Introduction

Metabolism is integral for health and diseases. It encapsulates events in living organisms that lead to the generation and utilization of energy for cellular processes [1]. Cancer cells manipulate metabolism to foster oncogenic transformations that enable aggressiveness for survival, proliferation, migration, invasion, and attachment to other sites resulting in metastatic tumors [2]. Multifaceted biochemical processes are involved in the metastatic cascade for the effectual dissemination of tumor cells. A major step in metastasis is the detachment of cells from one another and the extracellular matrix (ECM) to migrate and adhere to distal points [3]. Under normal circumstances, detaching cells are eradicated by programmed cell death termed anoikis [4]. In metastatic tumors, anoikis machinery is often overwhelmed by transformations such as epithelial-mesenchymal transition, metabolism reprogramming, and upregulation of pro-oncogenes. These transformations consequently facilitate cells adopting survival morphology that enable migration, invasion, and colonization of cancer cells in new sites [4,5,6]. Therefore, strategies to promote anoikis are fundamental to curb tumor metastasis.

The role of ECM detachment mediators (such as cell adhesion molecules, growth protein reactive oxygen species) modulating anoikis resistance and the metastatic cascade is yet to be fully demystified [5]. Changes in the metabolic process following detachment of tumor cells from ECM also modulate anoikis resistance [5,7,8], thus favoring oncogenic signals for survival in the host and disease progression. Altered metabolic activity is an important feature for tumor cells to successfully colonize distant sites. This colonization accounts for about 90% of cancer deaths [1,9].

ECM remodeling, deregulation of metabolic and signaling pathways are critical survival routes explored by detaching cells to escape anoikis stress [3,5]. Activation of PI3k, ERK/MAPK, among other signaling pathways modulate oncogenes, growth proteins and metabolites needed for anoikis resistance and metastasis [10]. ERK/MAPK axis is involved in enhanced proteolytic enzymes’ (metalloproteinase (MMP-2 and MMP-9) degradation and remodeling of the ECM [11]. ERK/MAPK signaling pathway was also reported to modulate RNF126 (growth related E3 ubiquitin ligase ring finger protein) activity in degrading pyruvate dehydrogenase kinases (PDK) [12]. Proteasome degradation of PDK led to enhanced generation of acetyl-CoA from pyruvate (catalyzed by pyruvate dehydrogenase). This resulted to a shift in metabolism from glycolysis to Krebs cycle, and aided alternate substrate availability for cells’ needs. ERK inhibition depleted growth related gene (RNF126) in both adherent and suspension conditions [12].

This study aims to unveil the relevance of circumventing transformations or events essential for cell detachment from the ECM that could potentiate anoikis resistance and metastasis. We are hopeful that this may lead to identifying new approaches to improve sensitivity to anoikis and metastasis prevention. To this end, cancer cells were cultured in low-adherent culture dishes using polyhema [13] to mimic biochemical and molecular changes inherent in ECM detaching cells, and if targeting such changes could promote tumor cells’ sensitivity to anoikis.

## 2. Materials and Methods

### 2.1. Reagent

Penicillin streptomycin (PS, SV30010), phosphate buffer saline (PBS, C10010500CP), were sourced from HYCLONE, (Logan, Utah, USA); DMEM medium (C11995050), trypsin EDTA (25200-56), and fetal bovine serum (FBS, A316081) were from Gibco (Waltham, MA, USA); chloromethyl-2, 7-dichlorofl-fluorescein diacetate (CM-H_2_DCFDA (Ref C6827)), mitosox red (Ref M36008), and Lipofectamine 3000 (L3000015) were procured from Invitrogen (Thermo Fisher Scientific, Waltham, MA, USA). Propidium iodide (PI) (Cat 421301) solution was purchased from Biolegend (San Diego, CA, USA). BCA Reagent A and B (23223 and 23224), poly-vinylidene fluoride (PVDF, ISEQ00010) from Millipore (County Cork, Ireland). Dorsomorphin 2HCl (Compound C) from Selleckchem (S7306). NP40 (P0013F), RIPA (P0013B) and SDS lysis buffer (P0013G), were from Beyotime (Shanghai, China). One-Step gDNA Removal and cDNA Synthesis SuperMix (AT311-03) sourced from TransGen (Beijing, China). Phosphatase inhibitors (04906845001) and protease inhibitors (11873580001) were from Roche (Basel, Switzerland). Enhanced chemiluminescence detection kit was obtained from Millipore (Burlington, MA, USA) (WBLUR0500). Standard culture plate from Corning incorporated (Corning, NY, USA) (12 plate: 3513; 6 plate: 3516 100 mm: 430167); Poly 2-hydroxyethyl methacrylate (Polyhema P3932), metformin, and β-actin (A5441, 1:10 dilution), were obtained from Sigma (St. Louis, MO, USA). K48-linkage specific polyubiquitin antibody ([EP8589] (ab140601, dilution 1:5) from Abcam, (Cambridge, UK), CHOP (L63F7 Mouse mAB, 2895S, dilution 1:1), AMPK (CST, 2532S, dilution 1:1), and p-AMPK (CST, PA5-36764, dilution 1:1) were from Cell Signaling Technology (Danvers, MA, USA), HRP-conjugated goat anti-rabbit IgG (E030120-02, dilution 1:10) from EARTHOX Millbrae, CA, USA), and HRP-conjugated goat anti-mouse IgG (074-1806, dilution 1:10) from KPL (Milford, MA, USA).

### 2.2. Cell Line

Cancer cell lines used in this study include human breast cancer (MCF-7), human cervical cancer (HeLa), and murine melanoma (B16F10). Cells were sourced from Shanghai cell bank, Chinese Academy of Sciences and were authenticated using short tandem repeat (STR) profiling, subculture and culture are carried out in a sufficiently clean environment with periodic mycoplasma test. All cell lines were cultured in DMEM supplemented with 1% PS, 10% FBS, in 37 °C, 5% CO_2_ humidified incubator Cells were cultured in standard culture dishes collected after passage, these denote adherent condition (0 h). Cells seeded in 20 mg/mL polyhema pre-coated dishes previously described [13] for 3, 6, 12, and 18 h denote anchorage-independent or suspension cells.

### 2.3. Transfection

Transfection of cancer cells with two validated AMPK α1 and α2 siRNA(s) was performed using Lipofectamine 3000 according to the manufacturer’s protocol. At 36 h post-transfection in standard culture plates, knockdown efficiency was confirmed by qRT-PCR. Subsequently, cells were seeded to a polyhema culture dish and treated with indicated drugs for 24 h. The specific sequences of the siRNA targeting human AMPK are 5′-GGAUUAUUGUCACAGGCAUTT-3′ (siAMPKα1, sense) and 5′-CCACUCUCCUGAUGCAUAUTT-3′ (siAMPKα2, sense).

### 2.4. Apoptosis Detection

The 1.0 × 10^5^ or 3.0 × 10^5^ cells/well of HeLa, MCF-7, or B16F10 seeded in triplicates in a 12 or 6-well polyhema culture dishes respectively were treated with indicated drugs and allowed to grow for 24 h. Cells were collected into tubes, centrifuged, trypsinized, washed with PBS, and stained with propidium iodide to acquire flow cytometer analysis. All samples were analyzed using CytoFLEX, flow cytometer from Beckman Coulter Life Sciences (Indianapolis, IN, USA)

### 2.5. Reactive Oxygen Species (ROS) Quantification

Mitotracker red was used to measure mitochondrial superoxide anion, while chloromethyl-2, 7-dichlorofluorescein diacetate (CM-H2DCFDA) was used to measure total or cellular ROS in HeLa, MCF-7, and B16F10 following treatment with specified drugs for 24 h in a 5% CO_2_ incubator. Cells were incubated away from light for 30 min at 37 °C and analyzed using the flow cytometer.

### 2.6. RNA-Seq

RNA from HeLa grown in standard culture dishes (0 h) and low adherent polyhema dish (suspension cells) at various time points (3, 6 12, and 18 h) was used for mRNA library preparation. Illumina HiSeq instrument was used to sequence the finalized libraries. Sequencing was performed with a 2 × 150 bp paired end (PE) configuration by GENEWIZ, Inc. (Suzhou, China). To classify the transformed pathways, RNA sequenced data were subjected to gene set enrichment analysis with the Broad Institute’s GSEA program, by employing KEGG gene sets from the Molecular Signature Database v.6.1. The normalized enrichment score (NES) was determined and analysed to assess the effects of all enrichment to identify individual gene sets that impact cells’ activities.

### 2.7. RNA Isolation and qRT-PCR

Trizol reagent was used for the extraction of total RNA, this was used to produce cDNA. Primers specific for each gene were synthesized by GENWIZ Corporation for quantitative real-time PCR analyses. The primer sequence is presented in Supplementary material Table S1, each sample was run in triplicate. Quantification was performed at least 3 independent times with similar results obtained.

### 2.8. Protein Extraction and Immunoblotting

Cells were lysed in NP-40 buffer on ice for 30 min and successively pelleted at 4 °C, for 15 (16,000 g). The supernatant was labeled as NP-40-soluble (NS) portion. The protein concentration in the NS portion was determined following BCA assay (Pierce, Rockford, IL, USA). The 2% SDS with 50 mM DTT (SDS buffer) was used to re-suspend the pellet designated SDS-soluble (SS). NS and SS fractions were analyzed by western blot.

The preparation of the whole-cell lysate for western blot analysis was performed in RIPA lysis buffer (Beyotime) augmented with 1× phosphatase inhibitor and 1× complete protease inhibitor (Roche). The protein concentration was measured using BCA assay. An equivalent quantity of protein was loaded and ran by molecular weight on SDS/PAGE, gel was transferred to polyvinylidene fluoride (PVDF) membrane, and blotted with corresponding protein antibodies. Proteins were viewed using the enhanced chemiluminescence detection kit (Millipore) on Amersham Imager 600 (GE Healthcare, Chicago, IL, USA).

### 2.9. Intracellular ATP Detection

Intracellular ATP was determined with Beyotime Biotechnology ATP assay kit following the product manual. Cells were prepared in lysis buffer provided and pelleted at 4 °C centrifuge for 5 min at 12,000× *g*. Supernatants were used for ATP detection. The 30 µL of supernatant were pipetted into room temperature preincubated 100 μL ATP detection working solution. Luminance (RLU) was measured using the Promega luminometer. Calculation of ATP concentration was performed with standard curve and normalized with the cells’ protein concentration.

### 2.10. Tumor Models

Six to eight weeks aged male BALB/c nude mice were procured from the Guangdong Medical Lab Animal Center (Guangzhou, China) and accommodated under pathogen free conditions in the animal facility of the Shenzhen Institute of Advanced Technology, Chinese Academy of Sciences. The experiment was designed and approved with due consideration to the 3Rs principle of animal use for research [14,15]. The anti-metastasis effects of three drugs were simultaneously evaluated using the same controls to reduce the number of animals to the minimum. However, the mechanisms and conclusion of studies differ which led to different publications [13].

Mice were inoculated intravenously with 0.5 × 10^6^ cells in 100 μL PBS. They were blindly divided to 3 treatments groups; control (PBS daily), PS341 (10 μg per mouse every 3 days), and metformin (5 mg per mice daily) injected intraperitoneally with their weight recorded. The study was repeated twice, and data pooled from independent experiments are presented.

### 2.11. Statistical Analysis

GraphPad Prism (6) Software (San Diego, CA, USA) was used for all data analysis. Data are expressed as mean ± SEM, one-way, or two-way ANOVA, Student’s *t*-test, with Tukey’s post-test were used for data analysis where *p* values < 0.05 were considered significant. In figures, level of significance were represented as: *, *p* ≤ 0.05; **, *p* ≤ 0.01; ***, *p* ≤ 0.001; and ****, *p* ≤ 0.0001.

## 3. Results

### 3.1. ECM Detachment Reprograms Metabolism in Tumor Cells

**Figure 1:**
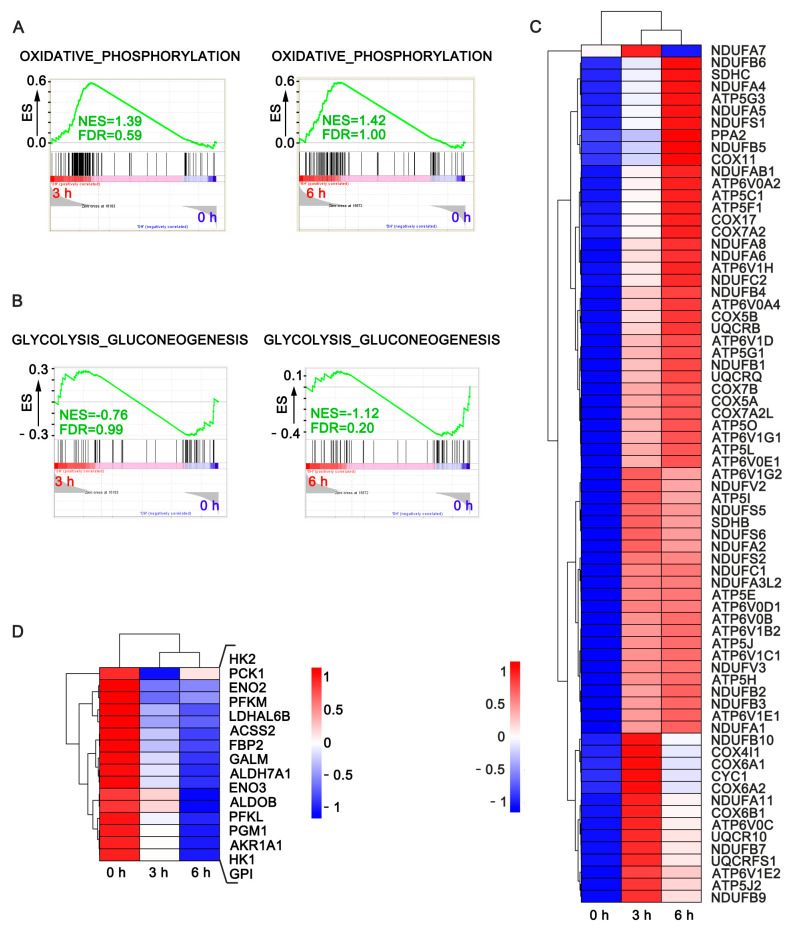
Metabolism reprogramming characteristic of cancer cells cultured in anchorage independent condition. (**A**) GSEA for the enrichment of oxidative phosphorylation-related genes in HeLa cultured in suspension for 3 and 6 h compared to the control (adherent cells, 0 h); (**B**) GSEA for the enrichment of glucose metabolism-related genes of HeLa in suspension following ECM-detachment after 3 and 6 h compared to the control (attached cell, 0 h); (**C**,**D**) heat maps show the expression patterns for the genes contributing to core enrichment of oxidative phosphorylation and glucose metabolism; the indicated pathway in HeLa cultured in suspension after 3 h of cell detachment and adherent cells at 0 h as control.

To investigate the underlying biochemical pathways mediating anoikis resistance in anchorage-independent cells, cervical cancer cells were cultured in normal dish collected post trypsin at 0 h and seeded in polyhema dishes for 3, 6, 12, and 18 h (Figure S1A). Cells were lysed with trizol for transcriptomics analysis to elucidate altered biochemical and molecular pathways. Our data revealed significant alterations in genes related to glucose, fatty acid and oxidative phosphorylation in cell-cultured in ECM detached conditions compared to the control at various time points as shown in the gene set enrichment analysis (Figure 1A,B). The heat maps show increased expression patterns for most genes contributing to core enrichment of oxidative phosphorylation (Figure 1C) and decreased expression of glucose metabolism and lipid synthesis (ACSS2) related pathways (Figure 1D).

### 3.2. Targeting Oxidative Phosphorylation by Metformin Promotes Anoikis in Cancer Cells

**Figure 2:**
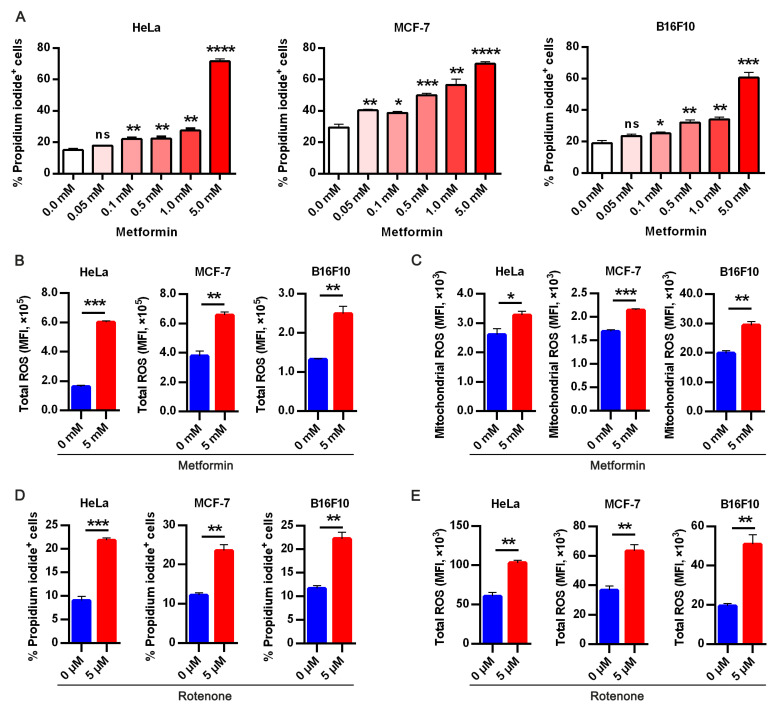
Targeting oxidative phosphorylation by metformin promotes anoikis in cancer cells. (**A**) Apoptotic HeLa, MCF-7, and B16F10 expressed as percentage propidium iodide + cells treated with indicated doses of metformin in ECM detached condition for 48 h; (**B**) flow cytometry analysis of 24 h metformin treatment on total cellular reactive oxygen species (ROS) measured as mean fluorescence intensity in HeLa, MCF-7, and B16F10 stained with CM-H2DCFDA (5 μM) for 30 min; (**C**) flow cytometry mean fluorescence intensity quantification of mitochondria superoxide anion in HeLa, MCF-7, and B16F10 cells following metformin treatment for 24 h; (**D**) flow cytometry assay of percentage propidium iodide + HeLa, MCF-7, and B16F10 treated with specific complex 1 inhibitor-rotenone (5 μM); (**E**) flow cytometry analysis of 5 μM CM-H2DCFDA stained HeLa, MCF-7 and B16F10 presented as mean fluorescence intensity in 24hours treatment with rotenone All result are mean SEM, (*n* = 3) * *p* < 0.05, ** *p* < 0.01, *** *p* < 0.001 **** *p* < 0.0001, ns, not significant; typical data from at least three independent experiments with similar results.

Based on the alterations observed in metabolic genes from the transcriptomics analysis, we investigated if metformin—a metabolic drug employed in the treatment of type 2 diabetes mellitus and also a mitochondrial Complex 1 inhibitor [16] could be repurposed to promote anoikis. We chose metformin treatment doses based on previous studies [17,18,19,20,21], interestingly, metformin significantly enhanced anoikis in all cell lines examined in a dose-dependent manner (Figure 2A and Figure S1B). Previous studies reported reactive oxygen species (ROS) involvement in the evasion and promotion of anoikis [13,22,23]. Based on metformin efficacy in sensitizing cells to anoikis in a dose-dependent manner, and considering a previous study reported its effect on mitochondria dysfunction [24]. We sought to know if metformin has an impact on ROS generation. Thus, we detected cellular and mitochondrial ROS generation in ECM detached cultured cells with or without metformin treatment. As shown in Figure 2B,C, metformin significantly increased cellular and mitochondria ROS.

Similarly, rotenone—a specific inhibitor of Complex 1 was used to ascertain metformin’s function and its effect on Complex 1 in promoting sensitivity to anoikis. As expected, rotenone elicited similar effects as it sensitized cells to anoikis (Figure 2D). Likewise, we sought to elucidate if rotenone affects intracellular ROS generated and we observed similar effects with metformin through the increased intracellular ROS generated in cancer cells (Figure 2E). These suggest increased mitochondria metabolic activity is one of the mechanisms by which cancer cells evade anoikis.

### 3.3. Metformin Abrogates Upregulated Proteasome Activity in ECM Detached Tumor Cells

**Figure 3:**
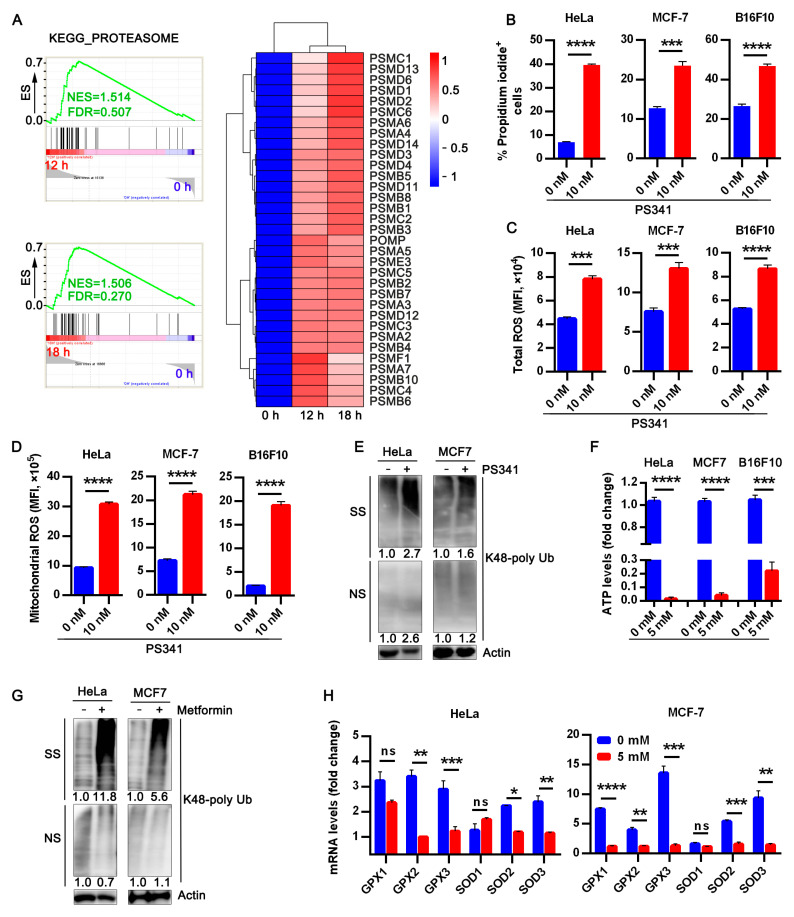
Metformin abrogates upregulated proteasome activity in ECM detached tumor cells. (**A**) GSEA for the enrichment of proteasome genes of HeLa in suspension at 12 and 18 h anchorage-independent growth compared to anchorage-dependent cells at 0 h. The heat map shows expression patterns for the genes contributing to core enrichment of proteasome activity; (**B**) flow cytometry assay data for percentage propidium iodide +HeLa, MCF-7, and B16F10 treated with 10 nM PS341 in ECM detached condition for 24 h; (**C**) flow cytometry data for total ROS quantification in tumor cells treated with PS341 measured as mean fluorescence intensity in cells stained with CM-H2DCFDA; (**D**) flow cytometry mean fluorescence intensity quantification of mitochondria ROS in HeLa, MCF-7, and B16F10 cells following PS341 treatment for 24 h; (**E**) Western blot of K48 polyUb-transformed proteins in 24 h PS341 untreated or treated detached HeLa and MCF-7, NS and SS portions are presented. NS—NP40 soluble portion, SS—SDS soluble portion. The uncropped western blot images of Figure 3E are in Supplementary material Figures S2 and S3; (**F**) luminance intracellular ATP level of metformin-treated cells presented as fold change relative to the controls; (**G**) blot of K48 polyUb-transformed proteins in 24 h metformin treated detached HeLa and MCF-7 cells, NS and SS portions are presented. NS—NP40 soluble portion, SS—SDS soluble portion. Uncropped western blot images of Figure 3G are provided in Supplementary Figures S4 and S5; (**H**) RT-qPCR relative mRNA level of antioxidant genes in HeLa and MCF-7 following treatment with metformin for 24 h. Data represent means ± SEM, (*n* = 3) * *p* < 0.05, ** *p* < 0.01, *** *p* < 0.001 **** *p* < 0.001 typical data from at least three independent experiments with similar results.

In addition to the changes in metabolic-related genes from our transcriptomics profiling data, we observed increased proteasome activity in anchorage-independent HeLa cells cultured for either 12 or 18 h (Figure 3A). qRT-PCR was utilized to ascertain the level of some proteasome genes in both anchorage-dependent and anchorage-independent HeLa and MCF-7 cells. Consistent with our RNA-seq data, we observed increased mRNA expression for evaluated proteasome genes in anchorage-independent cells compared to the control (Figure S1C).

Exposing cancer cells in suspension to specific proteasome inhibitor MG132 sensitized these cells to anoikis (Figure S1D). Previous studies reported a surge in ROS generation and cytotoxicity following proteasome inhibitors administration [25,26]. To this end, we investigated the impact of MG132 on intracellular and mitochondrial ROS production in anchorage-independent cells. As expected, there was increased ROS generation in treated groups compared to non-treated groups (Figure S1E,F). Next, we sought to assess if the FDA-approved proteasome inhibitor PS341 [27,28] elicits a similar effect as MG132 on cancer cells cultured in anchorage-independent conditions, Similarly, PS341 sensitized all tumor cells to anoikis (Figure 3B and Figure S1G), exhibited elevated cellular and mitochondrial ROS compared to untreated cells (Figure 3C,D).

The proteasome is reported to play critical roles in proteins degradation, metabolism, signal transduction, death, immunity, maintenance of cell quality control, and growth [29]. Next, we examined the impact of inhibiting proteasomes on protein clearance by evaluating K48 polyubiquitin aggregate, our result revealed accumulated misfolded protein in the proteasome inhibitor group compared to the control group (Figure 3E and Figure S1H) The uncropped western blot images of Figure S1H is presented in Figures S6 and S7.

Since either treatment with metformin or proteasome inhibitors promoted anoikis in B16F10, HeLa, and MCF-7, we imagined an interplay between OXPHOS and proteasome in impeding anoikis in cancer cells thereby fostering metastasis. Additionally, a previous study reported ATP is requisite for proteasome activity [30]. Since metformin inhibits OXPHOS, a major source of cellular ATP, we investigated the effect of metformin on ATP levels in these cells. Interestingly, metformin reduced the ATP level in these cancer cells (Figure 3F). In addition, we sought to evaluate the effect of metformin on the two consistently upregulated proteasome genes at various time points and found metformin could downregulate the mRNA expression of these genes (Figure S1I). Having observed increased ROS production following metformin treatment in these cells, and our finding based on the previous study from our group that elucidated interaction between oxidative stress and misfolded protein accumulation [13]. We proceeded to check K48 ubiquitin aggregation in metformin-treated cells and observed accumulation of misfolded protein in the metformin-treated group compared to the control (Figure 3G). Previous studies revealed superoxide dismutase (an antioxidant) activity facilitated anoikis resistance in ECM detached cancer cells [31,32,33], so we sought to investigate the effect of metformin treatment on two antioxidant families glutathione peroxidase 1–3 and superoxide dismutase 1–3 (Figure 3H). Our result showed metformin treatment significantly alleviated superoxide dismutase and glutathione peroxidase levels in tumor cells thus sensitizing these cells to anoikis. Therefore, OXPHOS-Proteasome interaction could be another path explored by cancer cells to circumvent anoikis and highlights the key role of metformin in modulating energy metabolism in ECM detached cells.

### 3.4. Metformin Treatment Activates AMPK and Apoptotic UPR Signaling Pathway

**Figure 4:**
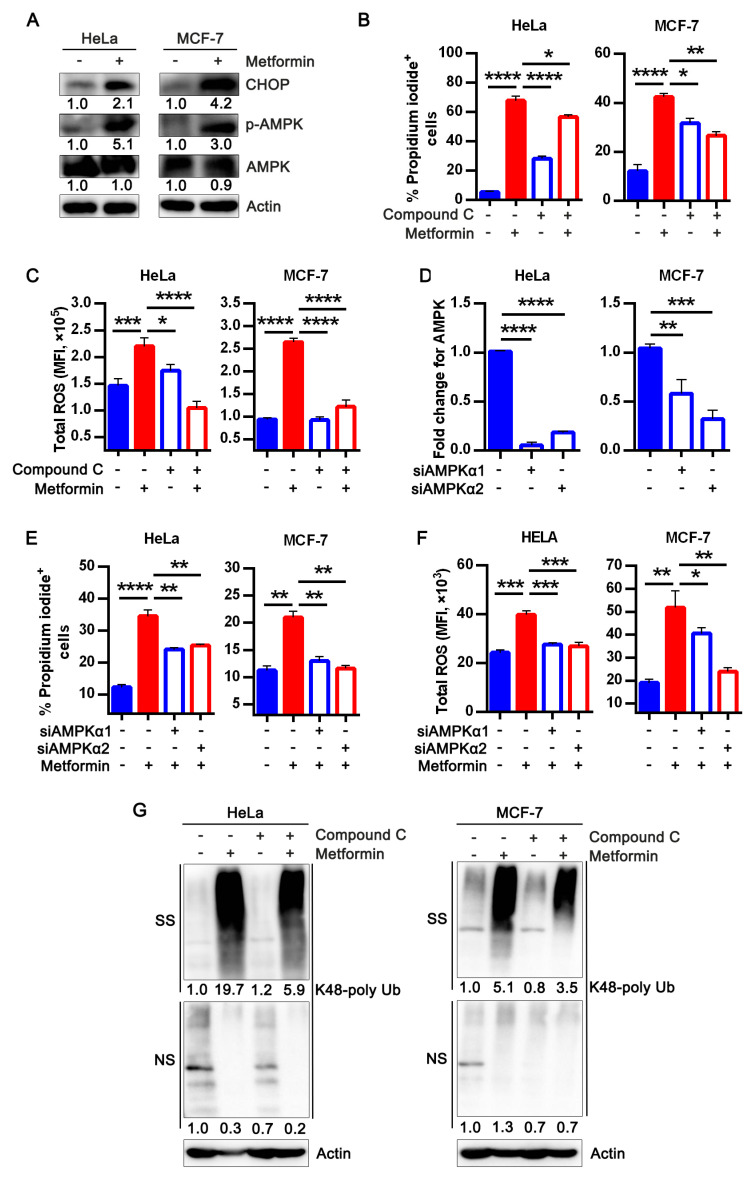
Metformin treatment activates AMPK and apoptotic UPR signaling pathway. (**A**) Lysates of HeLa and MCF-7 cells treated with or without metformin for 24 h evaluated for AMPK, p-AMPK, and CHOP and analyzed by immunoblotting. The original western blot images are provided in Supplementary material Figures S8 and S9; (**B**) percentage propidium iodide + HeLa and MCF-7 following treatment with or without 5 mM metformin in the presence or absence of 10 μM Compound C measured with flow cytometer; (**C**) quantification of total ROS measured as mean fluorescence intensity in metformin with/without Compound C treatment of HeLa and MCF-7stained with CM-H2DCFDA and measured with flow cytometer; (**D**) mRNA expression of AMPK α1 and α2 expressed as fold change relative to the controls transiently silenced in HeLa and MCF-7; (**E**) percentage propidium iodide + transiently silenced AMPK α1 and α2 cells (HeLa and MCF-7) 24 h treatment with/without metformin s and evaluated by flow cytometry while untreated cells serve as the control; (**F**) mean fluorescence intensity for ROS of transiently silenced AMPK α1 and α2 cells (HeLa and MCF-7) treatment with/without metformin for a period of 24 h while untreated cells serve as the control, stained for 30 min with CM-H2DCFDA kept from light and measured with flow cytometer; (**G**) blot of K48 polyUb-transformed proteins of HeLa and MCF-7 in suspension treated with metformin together with/without Compound C for 24 h, NS and SS portions are presented. NS—NP40 soluble portion, SS—SDS soluble portion. The uncropped western blot images are provided in Supplementary material Figures S10 and S11 Data analysis represents means ± SEM, (*n* = 3) * *p* < 0.05 ** *p* < 0.01, *** *p* < 0.001, **** *p* < 0.0001 typical data from at least three independent experiments with similar results.

AMPK is a metabolic regulator that maintains cellular energy homeostasis [34]. AMPK activation was reported as one of the mechanisms of metformin cytotoxicity [17,19]. However, AMPK activation in tumor cells is reported to be stress-induced as it senses changes in energy level to facilitate metabolic rewiring and homeostasis [35]. Subsequently, we evaluated if metformin-induced cell death via low ATP and enhanced ROS production in ECM detached cells is AMPK dependent. While there was no significant difference in total AMPK, we observed increased phosphorylation of AMPK in metformin-treated cells (Figure 4A). In addition, increased misfolded protein accumulation which suggests endoplasmic reticulum stress could be a path by which metformin elicited cytotoxicity. We investigated the pro-apoptotic transcription factor CHOP universally expressed in low concentration in physiological conditions and highly expressed during pro-apoptotic unfolded protein response resulting from stress [36]. Our protein analysis showed metformin increased CHOP expression (Figure 4A) which corroborates previous report that elucidated increased CHOP expression and stress resulted in reduced antioxidant level, and misfolded protein accumulation [37].

Next, we sought to know if impairing AMPK expression will affect metformin cytotoxic effects, then we treated cells with an AMPK inhibitor, Compound C in the presence or absence of metformin. We observed that Compound C protected cancer cells from metformin-induced cell death and stress (Figure 4B,C). In addition to pharmacological inhibition of AMPK, we silenced AMPK in HeLa and MCF-7 to validate the role of metformin-induced AMPK activation in promoting anoikis; confirmed its efficiency by evaluating AMPK mRNA expression (Figure 4D). Next, AMPK silenced cells were treated with or without metformin, we observed silencing AMPK protected cancer cells from metformin-induced cell death and reduced ROS generation in treated cells (Figure 4E,F) which further reveals the relevance of AMPK activation in propelling cytotoxic effects in cancer cells considered in this study.

Similarly, we examined the effect of Compound C with or without metformin on misfolded protein accumulation measured by K48 ubiquitin aggregate. We observed Compound C alone did not affect misfolded protein accumulation but reduced misfolded protein accumulation in combination with metformin (Figure 4G), signifying metformin-induced AMPK activation could contribute to misfolded protein accumulation.

### 3.5. Metformin or PS341 Impedes Lung Metastasis In Vivo

**Figure 5:**
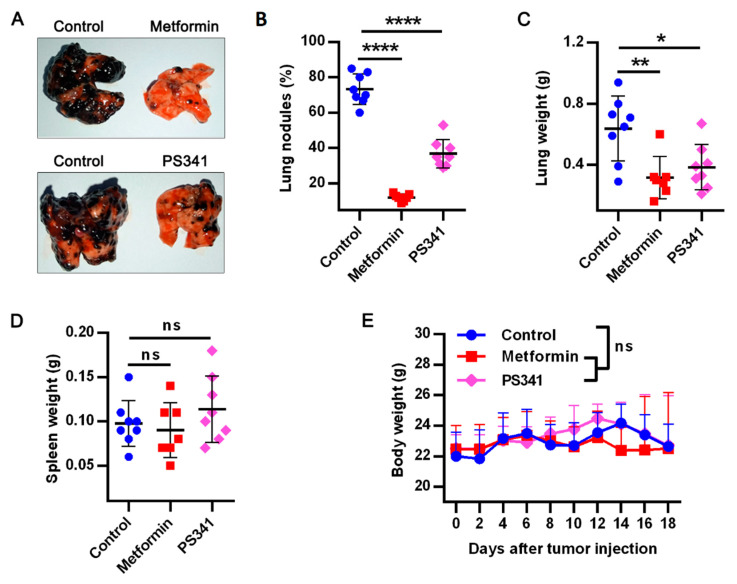
Metformin or PS341 impedes pulmonary metastasis in vivo. Balbc/nude male mice were injected intravenously with 0.5 × 10^6^ B16F10 and the tumor was allowed to grow for 18 days before sacrifice (n = 7–8 mice per group). The 5 mg metformin was administered to each mouse daily or 10 μg PS341 every 3 days starting from day 2 post tumor inoculation; (**A**) representative image of lungs excised from mice treated with metformin or PS341; (**B**) lung tumor nodules in mice treated with metformin or PS341; percentage nodules is calculated for each group as number of nodules on a lung ÷ highest number of nodules in the group ×100. (**C**) Lungs weight kinetics in both metformin and PS341 treatment; (**D**) spleen weight kinetics in both treatment group; (**E**) bodyweight kinetics of metformin and PS341 treated mice until Day 18 post tumor inoculation. Data for control group are similar to data presented in our previous publication [10,13] according to the 3R’s principle of animal use. Results presented as means ± SEM pooled from two independent investigations and analyzed with unpaired student *t*-test: * *p* < 0.05; ** *p* < 0.01; **** *p* < 0.0001; ns, not significant.

To partly confirm the in vitro effect of these therapy in vivo, we examined the effects of metformin or PS341 alone, on metastatic melanoma bearing balb/c nude mice, our study showed both drugs tremendously decreased pulmonary metastasis (Figure 5A), reduced tumor nodules (Figure 5B), and lung weight (Figure 5C). Notably, there were no changes in the spleen nor body weight of treated mice compared to control (Figure 5D,E), suggesting the safety of metformin and PS341 in being used to prevent metastasis.

## 4. Discussion

Despite the increasing knowledge in cancer treatment, metastasis remains a huge challenge to the clinical effects of current therapies. Cancer cells exploit metabolism, oncogenic signaling pathways to remodel ECM thus facilitate anoikis evasion, proliferation, and migration to distal points [1,5]. Cells in monolayer culture dishes are dispossessed of cell-cell and cell-ECM connections, they fail to depict the natural 3D morphology and molecular changes that occur in transformed cells in vivo [38]. Previous studies demonstrated the relevance of culturing cells in systems that imitate the 3D natural orientation in vitro. This enables effective study of the tumor environment, tumor interaction with ECM, transformations in tumor cells, nutrients availability, therapy design and testing [39,40,41].

Studies showed culturing cancer cells in polyhema coated dishes resulted in epithelial–messenchymal features switch from E-cadherin to N-cadherin, enhanced expression of oncogenic markers such as Slug, PI3K, AKT, ERK, EGFR, HER1 among other growth factors and receptors [42,43]. Here, we report metabolic transformations in cells cultured in suspension (to imitate early events in the metastatic cascade). Importantly transcriptional profiling of suspension or ECM detached cervical cancer cells revealed deregulated glucose, OXPHOS, and proteasome activity compared to the adherent cells. Thus, we used metformin or proteasome inhibitor(s) to target these pathways and found both significantly promote anoikis and suppress experimental metastasis.

Metformin an FDA-approved drug regulates glucose metabolism and inhibits complex 1, it is currently repurposed as an antineoplastic agent [16,17,19,44,45,46,47,48,49]. Similarly, proteasome inhibitors (MG132 and PS341) [27] were used to target proteasome upregulation in ECM detached cells. PS341 is also an FDA-approved drug for the treatment of multiple myeloma [28] but is currently explored in solid tumors [50,51]. Therefore, metformin and proteasome inhibitors (particularly PS341 approved for multiple myeloma treatment) promise as effective drug candidates for metastatic therapy.

Although, metformin promises as an anti-cancer agent [49,52], its mechanism of action in cancer of different origins is yet to be fully understood and whether it will be a potential therapy for metastasis—the leading cause of deaths in cancer [52,53]. Studies reported metformin had anticancer effect dependent or independent of AMPK activation [48,53]. AMPK activation was reported in various contexts to be involved in the modulation of stress and unfolded protein response (UPR) [54,55,56,57,58]. There are contrasting reports from studies using various cancer cells whether pro-apoptotic UPR recorded in metformin-treated cells is AMPK dependent or not [19,59]. However, whether activation of AMPK following metformin treatment in ECM detached cervical and breast cancer cells could foster pro-apoptotic UPR remains unknown.

Here we observe metformin exerts inhibitory effects on OXPHOS via reduced ATP level, increased cellular, and mitochondrial ROS, and cell death. This is similar to rotenone’s (specific complex I inhibitor) antineoplastic effects previously reported and also shown in our data [60,61]. This study reports metformin or PS341 sensitized anchorage-independent cancer cells to death via increased cellular and mitochondrial stress and significantly impaired pulmonary metastasis.

Metformin and PS341 at concentrations previously reported to induce cell death in attached cells [62,63] demonstrated similar effect in detached cells and highlight their potential to inhibit anoikis resistance in vitro. However, in vivo effect in other animal models [64] could result from drug interaction with immune cells [65], stroma, metabolic and signaling pathways to elicit antitumor effects. PS341 treatment repressed metastasis in hepatocellular and colorectal cancer by impeding EMT, invasiveness, and stemness [62]. PS341 with dendritic cell activated immune responses which led to reduced tumor burden [66]. Similarly, metformin treatment sensitized cancer cells to death, it cleared radiotherapy resistant cancer stem cells via AMPK activation [67]. Metformin was also reported to provoke immune response by reducing PD-L1 expression and synergizing with CD8 generated vaccine in triple negative breast cancer model [64].

**Figure 6:**
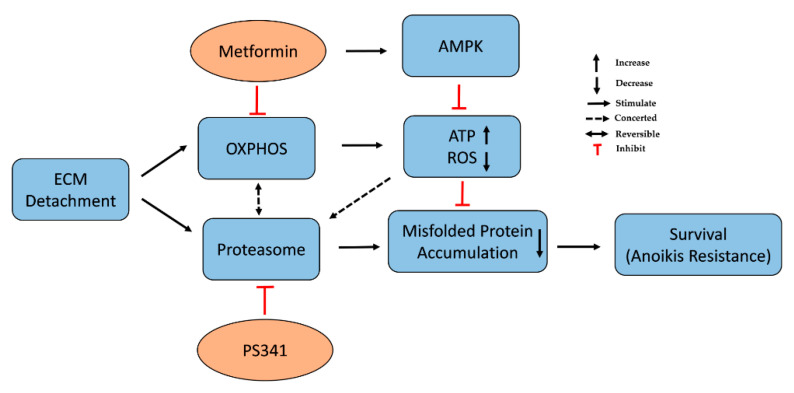
Targeting oxidative phosphorylation or proteasome activity with metformin or PS341 abrogated anoikis resistance and metastasis.

We report cellular energy sensor-AMPK (allosterically regulated by AMP/ADP: ATP level and stress) was activated in metformin-treated cells. This could be an attempt to preserve or restore mitochondrial metabolism for energy needs to promote survival or its activity as a tumor suppressor [68]. AMPK activation or depletion was reported to promote anoikis and, cell death [17,19,69]. Nonetheless, our data indicate AMPK activation by metformin exerted cytotoxic effects consistent with previous studies [17,19]. AMPK knockdown followed by metformin abrogated stress and cytotoxicity induced by metformin treatment alone. Here, metformin treatment in human breast (MCF-7), cervical cancer (HeLa), and murine melanoma (B16F10) inhibited anoikis resistance, via reduced ATP, increased cellular stress (Figure 6), and the expression of CHOP-transcriptional factor inherent in pro-apoptotic UPR and misfolded protein accumulation. This corroborates changes in metabolic activity is one of the mechanisms by which cancer cells evade anoikis. More so, metformin downregulated the expression of two highly expressed proteasome genes (PSMB2 and PSMD3) and abrogated antioxidant genes in ECM detached cells. Metformin modulates energy metabolism to induce its therapeutic effect in various disease conditions [53]. The observed reduction in ATP generated due to inhibition of complex I /OXPHOS fosters increased ROS generation and activation of AMPK in metformin-treated cells suggesting impaired mitochondrial metabolic function. This mitochondrial dysfunction impacts the ubiquitin-proteasome system’s ability to degrade proteins because of reliance on ATP. This led to the depletion of proteasome activity consistent with previous studies that reported mitochondrial energy metabolism modulates the proteasome system and vice versa [70,71,72].

Herein, metformin induced cellular stress, culminated in pro-apoptotic UPR via enhanced expression of CHOP and an increased misfolded protein accumulation. This is in line with our recent study on V-ATPase where we reported ROS generation and misfolded protein accumulation following V-ATPase inhibition sensitized cancer cells to anoikis [13]. Previously, tunicamycin treatment induced ER-stress and cytotoxicity via increased CHOP expression and impaired antioxidant activity. However pretreating cells with quercetin (an antioxidant) ameliorated tunicamycin induced stress and cytoxicity by impairing CHOP expression and normalizing antioxidant property [37]. Here, we report depletion in antioxidant genes expression following stress induction and pro-apoptotic UPR effects of metformin, which support previous reports that antioxidants activity enhanced anoikis resistance [31,32,33,73].

Importantly, pharmacological inhibition or genetic ablation of AMPK protected ECM detached cells from metformin-induced stress and anoikis in cervical and breast cancer cell lines. Our data show decrease in misfolded protein accumulation on treatment with metformin and AMPK inhibitor (Compound C), this confirms metformin elicited antitumor effect in these cancer cells in an AMPK-dependent manner to stimulate UPR in line with the study of Leclerc et al., [19]. Although a recent study by Conza et al., reported metformin impaired unfolded protein response in an AMPK independent manner in endometrial cancer cells, this discrepancy could be as a result of cancer cells of different origins [59].

Our study shows targeting proteasome activity enhanced cellular stress in line with Lipchick et al., [25], and increased misfolded protein accumulation which sensitized cells to anoikis. In addition, we demonstrated metformin’s ability to inhibit upregulated OXPHOS and proteasome activity via increased ROS generation, misfolded protein accumulation, depleted antioxidants, and proteasome activity, and thus, promote sensitivity to anoikis in cancer cells of varying origin (Figure 6).

A previous study on fibroblast reported metformin treatment reduced 26S proteasome activity. This supports our findings on its effect on proteasome activity in solid tumors; although these authors found that metformin confers increased resistance to PS341 [72]. On the contrary, metformin enhanced the anti-myeloma effect of PS341 by suppressing GRP78 autophagy and anti-apoptotic UPR-facilitators of protein homeostasis and potentiated relapse in PS341 lone treatment of multiple myeloma [74]. Importantly, in our study, metformin or PS341 treatment impaired pulmonary metastasis in the B16F10 melanoma model while metformin showed a more profound effect compared to PS341. In our future studies, we plan to examine the in vitro effects of these drugs in vivo and investigate if combining metformin and PS341 could demonstrate synergistic anti-tumor effect on solid tumors.

In conclusion, besides our knowledge of the antineoplastic effect of metformin on cancer cells, we discovered metformin treatment activated AMPK/CHOP expression that led to reduction in antioxidant activities and misfolded protein accumulation in ECM detached cells for the first time. Therefore, metformin can be repurposed to target abnormal metabolic reprogramming and proteasome activity responsible for anoikis resistance—a requisite for metastasis.

## Data Availability

The data that support the findings of this study are available from the corresponding authors upon reasonable request.

## Supplementary Materials

The following are available online at https://www.mdpi.com/article/10.3390/life12010042/s1, Table S1: Primer sequences for the quantitative PCR. Figure S1: Proteasome inhibition sensitizes tumor cells to anoikis, Figure S2: The original western blot for Figure 3E lysate of HeLa treated with PS341, showing Poly Ub K48, Figure S3: The uncropped western blot images for Figure 3E lysate of MCF-7 treated with PS341, showing Poly Ub K48; Figure S4: The original western blot for Figure 3G lysate of HeLa treated with metformin, showing Poly Ub K48 and Actin, Figure S5: The uncropped western blot images for Figure 3G lysate of MCF-7 treated with metformin, showing Poly Ub K48 and Actin, Figure S6: The uncropped western blot and of Suppl Figure S1E lysate of HeLa treated with MG132, showing Poly Ub K48 and Actin, Figure S7: The uncropped western blot images of Suppl Figure S1E lysate of MCF-7 treated with MG132, showing Poly Ub K48 and Actin, Figure S8: The original western blot images for Figure 4A, lysate of HeLa treated with metformin; CHOP, P-AMPK, AMPK and Actin, Figure S9: The uncropped western blot images for Figure 4A, lysate of MCF-7 treated with metformin; CHOP, P-AMPK, AMPK and Actin, Figure S10: The original western blot images for Figure 4G lysate of HeLa treated with metformin with or without compound C, showing Poly Ub K48 and Actin, Figure S11: The original western blot images for Figure 4G lysate of MCF-7 treated with metformin with or without compound C, showing Poly Ub K48 and Actin.

## References

[1] DeBerardinis R.J., Thompson C.B. (2012). Cellular metabolism and disease: What do metabolic outliers teach us?. Cell.

[2] Vander Heiden M.G., DeBerardinis R.J. (2017). Understanding the Intersections between Metabolism and Cancer Biology. Cell.

[3] Paoli P., Giannoni E., Chiarugi P. (2013). Anoikis molecular pathways and its role in cancer progression. Biochim. Biophys. Acta.

[4] Taddei M.L., Giannoni E., Fiaschi T., Chiarugi P. (2012). Anoikis: An emerging hallmark in health and diseases. J. Pathol..

[5] Adeshakin F.O., Adeshakin A.O., Afolabi L.O., Yan D., Zhang G., Wan X. (2021). Mechanisms for Modulating Anoikis Resistance in Cancer and the Relevance of Metabolic Reprogramming. Front. Oncol..

[6] Kim Y.-N., Koo K.H., Sung J.Y., Yun U.-J., Kim H. (2012). Anoikis Resistance: An Essential Prerequisite for Tumor Metastasis. Int. J. Cell Biol..

[7] Kamarajugadda S., Stemboroski L., Cai Q., Simpson N.E., Nayak S., Tan M., Lu J. (2012). Glucose oxidation modulates anoikis and tumor metastasis. Mol. Cell. Biol..

[8] Palorini R., Votta G., Pirola Y., De Vitto H., De Palma S., Airoldi C., Vasso M., Ricciardiello F., Lombardi P.P., Cirulli C. (2016). Protein Kinase: A Activation Promotes Cancer Cell Resistance to Glucose Starvation and Anoikis. PLoS Genet..

[9] Dillekås H., Rogers M.S., Straume O. (2019). Are 90% of deaths from cancer caused by metastases?. Cancer Med..

[10] de Sousa Mesquita A.P., de Araújo Lopes S., Pernambuco Filho P.C.A., Nader H.B., Lopes C.C. (2017). Acquisition of anoikis resistance promotes alterations in the Ras/ERK and PI3K/Akt signaling pathways and matrix remodeling in endothelial cells. Apoptosis Int. J. Program. Cell Death.

[11] Moulik S., Pal S., Biswas J., Chatterjee A. (2014). Role of ERK in Modulating MMP 2 and MMP 9 with Respect to Tumour Invasiveness in Human Cancer Cell Line MCF-7 and MDA-MB-231. J. Tumor.

[12] Yoshino S., Hara T., Nakaoka H.J., Kanamori A., Murakami Y., Seiki M., Sakamoto T. (2016). The ERK signaling target RNF126 regulates anoikis resistance in cancer cells by changing the mitochondrial metabolic flux. Cell Discov..

[13] Adeshakin F.O., Adeshakin A.O., Liu Z., Lu X., Cheng J., Zhang P., Yan D., Zhang G., Wan X. (2021). Upregulation of V-ATPase by STAT3 Activation Promotes Anoikis Resistance and Tumor Metastasis. J. Cancer.

[14] Hubrecht R.C., Carter E. (2019). The 3Rs and Humane Experimental Technique: Implementing Change. Animals.

[15] Graham M.L., Prescott M.J. (2015). The multifactorial role of the 3Rs in shifting the harm-benefit analysis in animal models of disease. Eur. J. Pharmacol..

[16] Wheaton W.W., Weinberg S.E., Hamanaka R.B., Soberanes S., Sullivan L.B., Anso E., Glasauer A., Dufour E., Mutlu G.M., Budigner G.R.S. (2014). Metformin inhibits mitochondrial complex I of cancer cells to reduce tumorigenesis. eLife.

[17] Saengboonmee C., Seubwal W., Cha’on U., Sawanyawisuth K., Wongkham S., Wongkham C. (2017). Metformin Exerts Antiproliferative and Anti-metastatic Effects Against Cholangiocarcinoma Cells by Targeting STAT3 and NF-ĸB. Anticancer Res..

[18] Davies G., Lobanova L., Dawicki W., Groot G., Gordon J.R., Bowen M., Harkness T., Arnason T. (2017). Metformin inhibits the development, and promotes the resensitization, of treatment-resistant breast cancer. PLoS ONE.

[19] Leclerc G.M., Leclerc J.G., Kuznetsov J.N., DeSalvo J., Barredo J.C. (2013). Metformin induces apoptosis through AMPK-dependent inhibition of UPR signaling in ALL lymphoblasts. PLoS ONE.

[20] De Santi M., Baldelli G., Diotallevi A., Galluzzi L., Schiavano G.F., Brandi G. (2019). Metformin prevents cell tumorigenesis through autophagy-related cell death. Sci. Rep..

[21] Wu B., Li S., Sheng L., Zhu J., Gu L., Shen H., La D., Hambly B.D., Bao S., Di W. (2012). Metformin inhibits the development and metastasis of ovarian cancer. Oncol. Rep..

[22] Li A.E., Ito H., Rovira I.I., Kim K.-S., Takeda K., Yu Z.-Y., Ferrans V.J., Finkel T. (1999). A Role for Reactive Oxygen Species in Endothelial Cell Anoikis. Circ. Res..

[23] Giannoni E., Buricchi F., Grimaldi G., Parri M., Cialdai F., Taddei M.L., Raugei G., Ramponi G., Chiarugi P. (2008). Redox regulation of anoikis: Reactive oxygen species as essential mediators of cell survival. Cell Death Differ..

[24] Yang M., Darwish T., Larraufie P., Rimmington D., Cimino I., Goldspink D.A., Jenkins B., Koulman A., Brighton C.A., Ma M. (2021). Inhibition of mitochondrial function by metformin increases glucose uptake, glycolysis and GDF-15 release from intestinal cells. Sci. Rep..

[25] Lipchick B.C., Fink E.E., Nikiforov M.A. (2016). Oxidative stress and proteasome inhibitors in multiple myeloma. Pharmacol. Res..

[26] Maharjan S., Oku M., Tsuda M., Hoseki J., Sakai Y. (2014). Mitochondrial impairment triggers cytosolic oxidative stress and cell death following proteasome inhibition. Sci. Rep..

[27] Kisselev A.F., van der Linden W.A., Overkleeft H.S. (2012). Proteasome inhibitors: An expanding army attacking a unique target. Chem. Biol..

[28] Chen D., Frezza M., Schmitt S., Kanwar J., Dou Q.P.L. (2011). Bortezomib as the first proteasome inhibitor anticancer drug: Current status and future perspectives. Curr. Cancer Drug Targets.

[29] Muñoz C., Francisco J.S., Gutiérrez B., González J. (2015). Role of the Ubiquitin-Proteasome Systems in the Biology and Virulence of Protozoan Parasites. BioMed Res. Int..

[30] Peth A., Uchiki T., Goldberg A.L. (2010). ATP-dependent steps in the binding of ubiquitin conjugates to the 26S proteasome that commit to degradation. Mol. Cell.

[31] Li S., Mao Y., Zhou T., Luo C., Xie J., Qi W., Yang Z., Ma J.X., Gao G., Yang X. (2016). Manganese superoxide dismutase mediates anoikis resistance and tumor metastasis in nasopharyngeal carcinoma. Oncotarget.

[32] Kamarajugadda S., Cai Q., Chen H., Nayak S., Zhu J., He M., Jin Y., Zhang Y., Ai L., Martin S.S. (2013). Manganese superoxide dismutase promotes anoikis resistance and tumor metastasis. Cell Death Dis..

[33] Sousa B., Pereira J., Marques R., Grilo L.F., Pereira S.P., Sardão V.A., Schmitt F., Oliveira P.J., Paredes J. (2020). P-cadherin induces anoikis-resistance of matrix-detached breast cancer cells by promoting pentose phosphate pathway and decreasing oxidative stress. Biochim. Biophys. Acta Mol. Basis Dis..

[34] Hardie D.G. (2011). AMP-activated protein kinase: An energy sensor that regulates all aspects of cell function. Genes Dev..

[35] Zhao Y., Hu X., Liu Y., Dong S., Wen Z., He W., Zhang S., Huang Q., Shi M. (2017). ROS signaling under metabolic stress: Cross-talk between AMPK and AKT pathway. Mol. Cancer.

[36] Hu H., Tian M., Ding C., Yu S. (2019). The C/EBP Homologous Protein (CHOP) Transcription Factor Functions in Endoplasmic Reticulum Stress-Induced Apoptosis and Microbial Infection. Front. Immunol..

[37] Suganya N., Bhakkiyalakshmi E., Suriyanarayanan S., Paulmurugan R., Ramkumar K.M. (2014). Quercetin ameliorates tunicamycin-induced endoplasmic reticulum stress in endothelial cells. Cell Prolif..

[38] Kapałczyńska M., Kolenda T., Przybyła W., Zajączkowska M., Teresiak A., Filas V., Ibbs M., Bliźniak R., Łuczewski L., Lamperska K. (2018). 2D and 3D cell cultures—A comparison of different types of cancer cell cultures. Arch. Med. Sci..

[39] Breslin S., O’Driscoll L. (2016). The relevance of using 3D cell cultures, in addition to 2D monolayer cultures, when evaluating breast cancer drug sensitivity and resistance. Oncotarget.

[40] Chen Y.-C., Lou X., Zhang Z., Ingram P., Yoon E. (2015). High-Throughput Cancer Cell Sphere Formation for Characterizing the Efficacy of Photo Dynamic Therapy in 3D Cell Cultures. Sci. Rep..

[41] Lawrenson K., Grun B., Gayther S.A. (2012). Heterotypic three-dimensional in vitro modeling of stromal-epithelial interactions during ovarian cancer initiation and progression. J. Vis. Exp..

[42] Lim W.-C., Kim H., Kim Y.-J., Jeon B.-M., Kang H.-B., Ko H. (2020). Catechol inhibits epidermal growth factor-induced epithelial-to-mesenchymal transition and stem cell-like properties in hepatocellular carcinoma cells. Sci. Rep..

[43] Chunhacha P., Sriuranpong V., Chanvorachote P. (2013). Epithelial-mesenchymal transition mediates anoikis resistance and enhances invasion in pleural effusion-derived human lung cancer cells. Oncol. Lett..

[44] Zhang Z., Zhou L., Xie N., Nice E.C., Zhang T., Cui Y., Huang C. (2020). Overcoming cancer therapeutic bottleneck by drug repurposing. Signal Transduct. Target. Ther..

[45] Bailey C.J. (2017). Metformin: Historical overview. Diabetologia.

[46] Ko Y., Choi A., Lee M., Lee J.A. (2016). Metformin displays in vitro and in vivo antitumor effect against osteosarcoma. Korean J. Pediatr..

[47] Amable G., Martínez-León E., Picco M.E., Di Siervi N., Davio C., Rozengurt E., Rey O.L. (2019). Metformin inhibits β-catenin phosphorylation on Ser-552 through an AMPK/PI3K/Akt pathway in colorectal cancer cells. Int. J. Biochem. Cell Biol..

[48] Saini N., Yang X. (2017). Metformin as an anti-cancer agent: Actions and mechanisms targeting cancer stem cells. Acta Biochim. Biophys. Sin..

[49] Adeshakin F.O., Zhang G., Adeshakin A.O., Wan X. (2021). Abstract 2869, Blockade of oxidative phosphorylation by metformin promotes anoikis. Cancer Res..

[50] Huang Z., Wu Y., Zhou X., Xu J., Zhu W., Shu Y., Liu P. (2014). Efficacy of therapy with bortezomib in solid tumors: A review based on 32 clinical trials. Future Oncol..

[51] Roeten M.S.F., Cloos J., Jansen G. (2018). Positioning of proteasome inhibitors in therapy of solid malignancies. Cancer Chemother. Pharmacol..

[52] Aljofan M., Riethmacher D. (2019). Anticancer activity of metformin: A systematic review of the literature. Future Sci. OA.

[53] Yan Y., Kover K.L., Moore W.V. (2020). New Insight into Metformin Mechanism of Action and Clinical Application.

[54] Amodio G., Moltedo O., Faraonio R., Remondelli P. (2018). Targeting the Endoplasmic Reticulum Unfolded Protein Response to Counteract the Oxidative Stress-Induced Endothelial Dysfunction. Oxidative Med. Cell. Longev..

[55] Yang L., Sha H., Davisson R.L., Qi L. (2013). Phenformin Activates the Unfolded Protein Response in an AMP-activated Protein Kinase (AMPK)-dependent Manner. J. Biol. Chem..

[56] Meares G.P., Hughes K.J., Naatz A., Papa F.R., Urano F., Hansen P.A., Benveniste E.N., Corbett J.A. (2011). IRE1-dependent activation of AMPK in response to nitric oxide. Mol. Cell. Biol..

[57] Kimura Y., Irie K., Mizuno T. (2017). Expression control of the AMPK regulatory subunit and its functional significance in yeast ER stress response. Sci. Rep..

[58] Zimmermann K., Baldinger J., Mayerhofer B., Atanasov A.G., Dirsch V.M., Heiss E.H. (2015). Activated AMPK boosts the Nrf2/HO-1 signaling axis—A role for the unfolded protein response. Free. Radic. Biol. Med..

[59] Conza D., Mirra P., Calì G., Insabato L., Fiory F., Beguinot F., Ulianich L. (2021). Metformin Dysregulates the Unfolded Protein Response and the WNT/β-Catenin Pathway in Endometrial Cancer Cells through an AMPK-Independent Mechanism. Cells.

[60] Del Prete A., Zaccagnino P., Di Paola M., Saltarella M., Celis C.O., Nico B., Santoro G., Lorusso M. (2008). Role of mitochondria and reactive oxygen species in dendritic cell differentiation and functions. Free Radic. Biol. Med..

[61] Li N., Ragheb K., Lawler G., Sturgis J., Rajwa B., Melendez J.A., Robinson J.P. (2003). Mitochondrial Complex I Inhibitor Rotenone Induces Apoptosis through Enhancing Mitochondrial Reactive Oxygen Species Production. J. Biol. Chem..

[62] Yang Z., Liu S., Zhu M., Zhang H., Wang J., Xu Q., Lin K., Zhou X., Tao M., Li C. (2016). PS341 inhibits hepatocellular and colorectal cancer cells through the FOXO3/CTNNB1 signaling pathway. Sci. Rep..

[63] Yamashita T., Kato K., Fujihara S., Iwama H., Morishita A., Yamana H., Kobayashi K., Kamada H., Chiyo T., Kobara H. (2020). Anti-diabetic drug metformin inhibits cell proliferation and tumor growth in gallbladder cancer via G0/G1 cell cycle arrest. Anti-Cancer Drugs.

[64] Munoz L.E., Huang H.L., Guin R.N., Bommireddy R., Selvaraj P. (2020). Metformin reduces PD-L1 expression in the tumor and enhances the efficacy of vaccine generated CD8 T cells in a murine model of triple negative breast cancer. J. Immunol..

[65] Bahrambeigi S., Shafiei-Irannejad V. (2020). Immune-mediated anti-tumor effects of metformin; targeting metabolic reprogramming of T cells as a new possible mechanism for anti-cancer effects of metformin. Biochem. Pharmacol..

[66] Schumacher L.Y., Vo D.D., Garban H.J., Comin-Anduix B., Owens S.K., Dissette V.B., Glaspy J.A., McBride W.H., Bonavida B., Economou J.S. (2006). Immunosensitization of Tumor Cells to Dendritic Cell-Activated Immune Responses with the Proteasome Inhibitor Bortezomib (PS-341, Velcade). J. Immunol..

[67] Song C.W., Lee H., Dings R.P.M., Williams B., Powers J., Santos T.D., Choi B.-H., Park H.J. (2012). Metformin kills and radiosensitizes cancer cells and preferentially kills cancer stem cells. Sci. Rep..

[68] Chuang H.-C., Chou C.-C., Kulp S.K., Chen C.-S. (2014). AMPK as a potential anticancer target—Friend or foe?. Curr. Pharm. Des..

[69] Guo P., Qiu Y., Ma X., Li T., Ma X., Zhu L., Lin Y., Han L. (2018). Tripartite motif 31 promotes resistance to anoikis of hepatocarcinoma cells through regulation of p53-AMPK axis. Exp. Cell Res..

[70] Krämer L., Groh C., Herrmann J.M. (2021). The proteasome: Friend and foe of mitochondrial biogenesis. FEBS Lett..

[71] Lavie J., De Belvalet H., Sonon S., Ion A.M., Dumon E., Melser S., Lacombe D., Dupuy J.-W., Lalou C., Bénard G. (2018). Ubiquitin-Dependent Degradation of Mitochondrial Proteins Regulates Energy Metabolism. Cell Rep..

[72] Meul T., Berschneider K., Schmitt S., Mayr C.H., Mattner L.F., Schiller H.B., Yazgili A.S., Wang X., Lukas C., Schlesser C. (2020). Mitochondrial Regulation of the 26S Proteasome. Cell Rep..

[73] Schafer Z.T., Grassian A.R., Song L., Jiang Z., Gerhart-Hines Z., Irie H.Y., Gao S., Puigserver P., Brugge J.S. (2009). Antioxidant and oncogene rescue of metabolic defects caused by loss of matrix attachment. Nature.

[74] Jagannathan S., Abdel-Malek M.A.Y., Malek E., Vad N., Latif T., Anderson K.C., Driscoll J.J. (2015). Pharmacologic screens reveal metformin that suppresses GRP78-dependent autophagy to enhance the anti-myeloma effect of bortezomib. Leukemia.

