# Supplementary material for: Targeting Oxidative Phosphorylation-Proteasome Activity in Extracellular Detached Cells Promotes Anoikis and Inhibits Metastasis
Source: Life (Basel). 2021 Dec 28;12(1):42. doi: 10.3390/life12010042 (PMC8779336; doi:10.3390/life12010042)
Supplement: Supplementary file 1 [file life-12-00042-s001.zip › life-1464196-supplementary.pdf]

Supplementary materials

Targeting Oxidative Phosphorylation-Proteasome Activity in Extracellular Detached Cells Promotes Anoikis and Inhibits Metastasis

Supplementary material

Table S1. Primer sequences for the quantitative PCR.

S/N	GENE	FORWARD-F AND REVERSE-R
1	PSMA4	F- TCCGTGGACATCTCAGGTCT<br>R- TGGTATAAGCGACCTTCTGGAG
2	PSMB2	F- ATCTTCGCTCTGAGGTGCTG<br>R- TTGTCATGATCGATGAGGTACTCC
3	PSMD1	F- GGCCAAGTTTGGCGCTATTC<br>R- TGAACTTTCGGCATCTTTAAGTCC
4	PSMD3	F- ACCTCTACTACACAGGGCGA<br>R- AACAGCTCCACCACGATGAG
4	AMPKα1	F- CTCCACGAAGGAGCTGGATG<br>R- TCTGGTGCAGCATAGTTGGG
5	AMPKα 2	F- TGAGAAGCAGAAGCACGACGG<br>R- AACTGCCACTTATGGCCTGTTA
6	GPX1	F- CCAGTTTGGGCATCAGGAGAA<br>R- AGCATGAAGTTGGGCTCGAA
7	GPX2	F- GACTTCACCCAGCTCAACGA<br>R- ATGCTCGTTCTGCCCATTCA
8	GPX3	F- AGAAGTCGAAGATGGACTGCC<br>R- GCAGGGAAAGCCCAGAATGA
9	SOD1	F- GCAGGGAAAGCCCAGAATGA<br>R- AACGACTTCCAGCGTTTCCT
10	SOD2	F- GGCCTACGTGAACAACCTGA<br>R- GTTCTCCACCACCGTTAGGG
11	SOD3	F- GATCCGAGACATGTACGCCA<br>R- GAAGAAGGCGTCGAGCTTGG
12	B-Actin	F- ATTGGCAATGAGCGGTTCCG<br>R- AGGGCAGTGATCTCCTTCTG

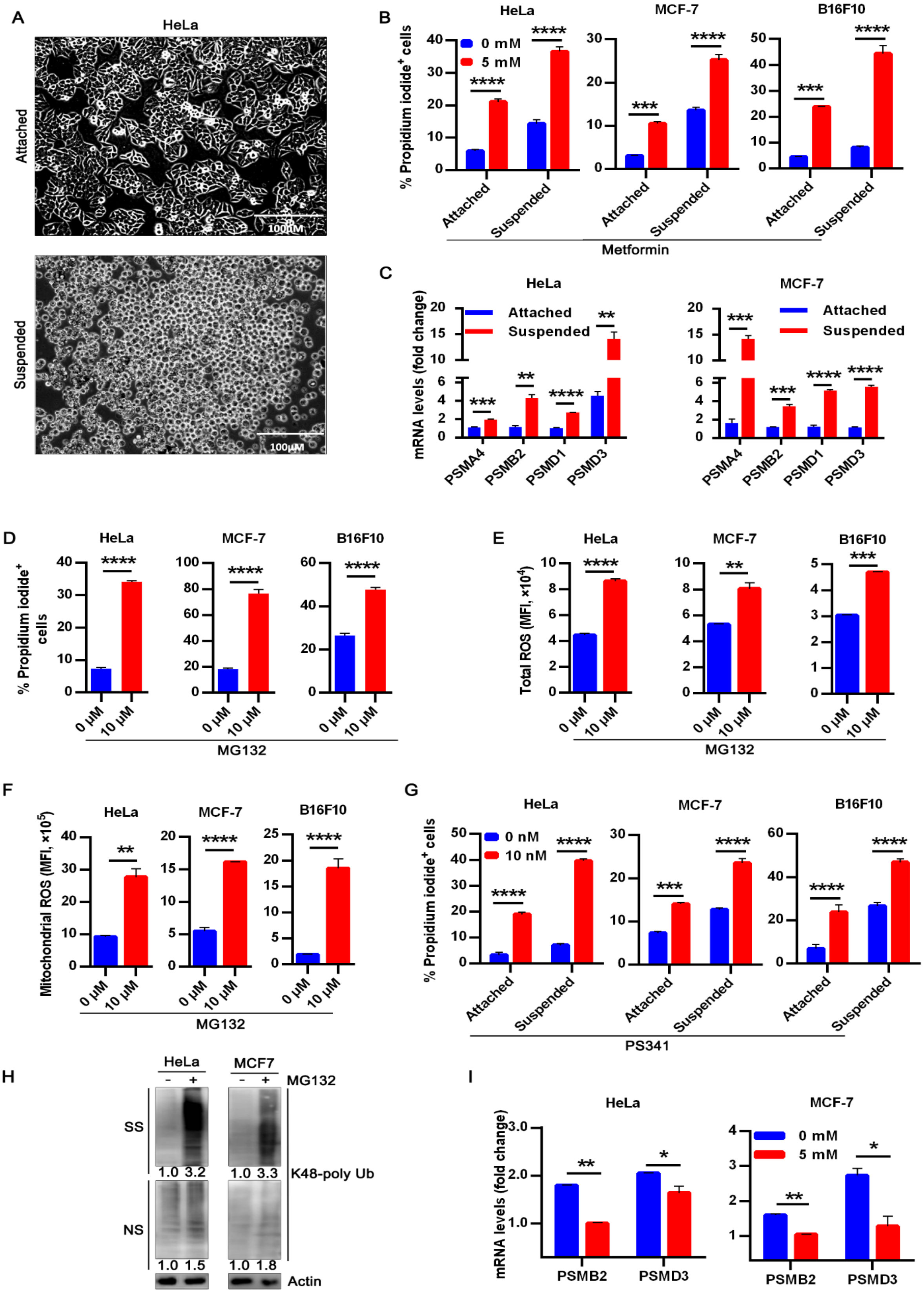
A
HeLa
Attached
Suspended
100μM
100μM
B
HeLa
MCF-7
B16F10
% Propidium iodide+ cells
0 mM
5 mM
Attached
Suspended
Metformin
C
HeLa
MCF-7
Attached
Suspended
mRNA levels (fold change)
PSMA4
PSMB2
PSMD1
PSMD3
D
HeLa
MCF-7
B16F10
% Propidium iodide+ cells
0 μM
10 μM
MG132
E
HeLa
MCF-7
B16F10
Total ROS (MFI, ×10⁴)
0 μM
10 μM
MG132
F
HeLa
MCF-7
B16F10
Mitochondrial ROS (MFI, ×10⁵)
0 μM
10 μM
MG132
G
HeLa
MCF-7
B16F10
% Propidium iodide+ cells
0 nM
10 nM
Attached
Suspended
PS341
H
HeLa
MCF7
MG132
SS
NS
1.0 3.2
1.0 3.3
1.0 1.5
1.0 1.8
K48-poly Ub
Actin
I
HeLa
MCF-7
mRNA levels (fold change)
0 mM
5 mM
PSMB2
PSMD3

Figure S1. Proteasome inhibition sensitizes tumor cells to anoikis. (A) Phase-contrast microscopic images of HELA cultured in normal (attached) and polyhema pre-coated (suspended) dishes; (B) Percentage apoptotic HeLa, MCF-7, and B16F10 treated with Metformin in ECM attached or detached condition; (C) RT-qPCR relative mRNA expression of proteasome genes in HeLa and MCF-7 cultured for 24 hours as adherent or suspension cells. (D) Flow cytometry percentage propidium iodide+ HeLa, MCF-7, and B16F10 treated with 10 μM MG132 in ECM detached condition for 24 hours. (E) Flow cytometry quantification of total ROS measured as mean fluorescence intensity in MG132 treatment of HeLa, MCF-7, and B16F10 stained with CM-H2DCFDA. (F) Flow cytometry mean fluorescence intensity quantification of mitochondria ROS in HeLa, MCF-7, and B16F10 cells after MG132 treatment for 24 hours. (G) Percentage apoptotic HeLa, MCF-7, and B16F10 treated with PS341 in ECM attached or detached condition. (H) Blot of K48 polyUb-transformed proteins in MG132 treatment of HeLa and MCF-7, NS and SS portions are presented. NS – NP40 soluble portion, SS – SDS soluble portion. (I) Effect of metformin treatment on the relative mRNA content of some proteasome genes after 24 hours of treatment of HeLa and MCF-7 measured by RT-qPCR. All result are mean SEM, (n=3), * $p < 0.05$, ** $p< 0.01$, *** $p < 0.001$ ****$p < 0.0001$, ns, not significant difference**.**

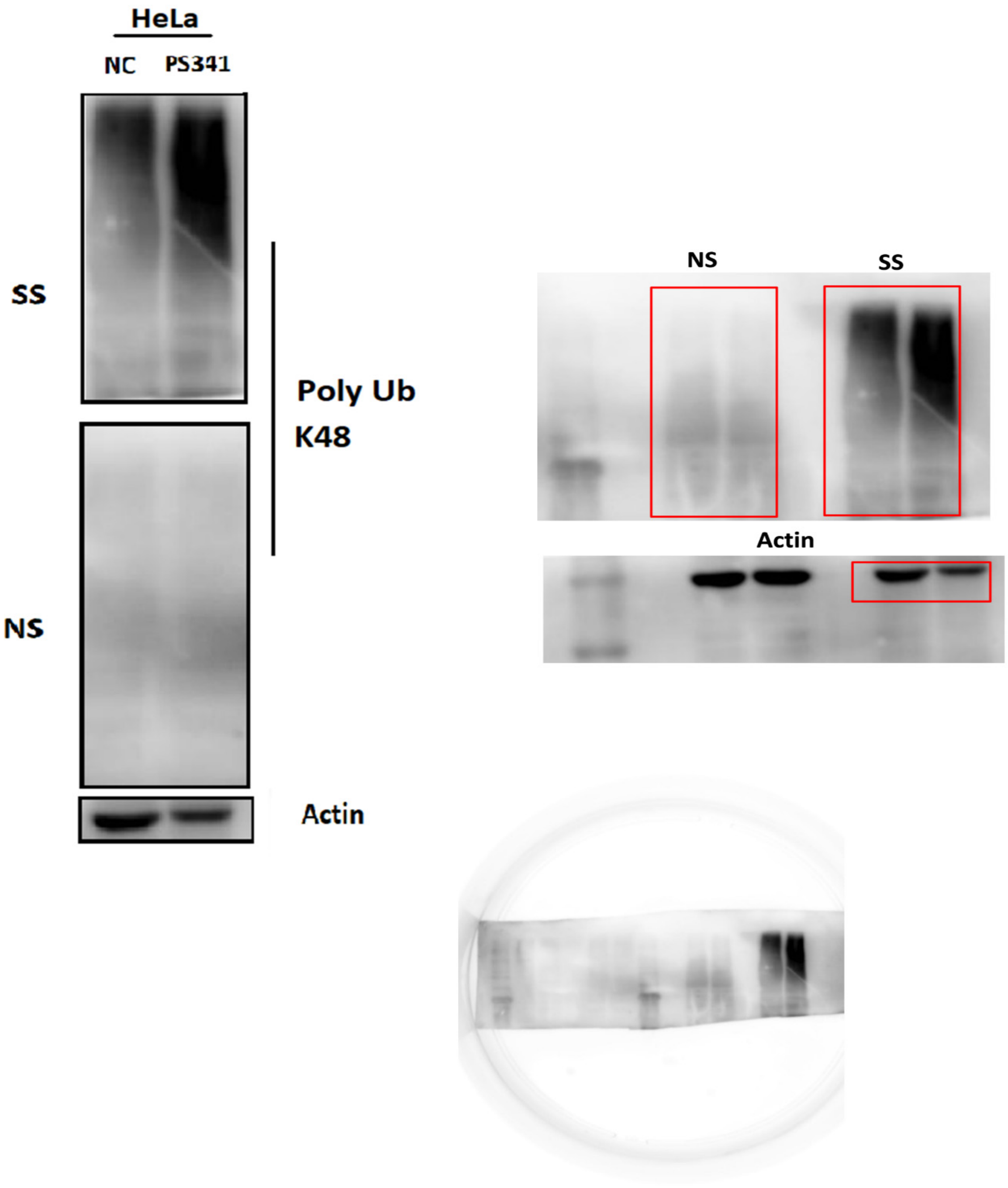

Figure S2. The original western blot for Figure 3E lysate of HeLa treated with PS341, showing Poly Ub K48.

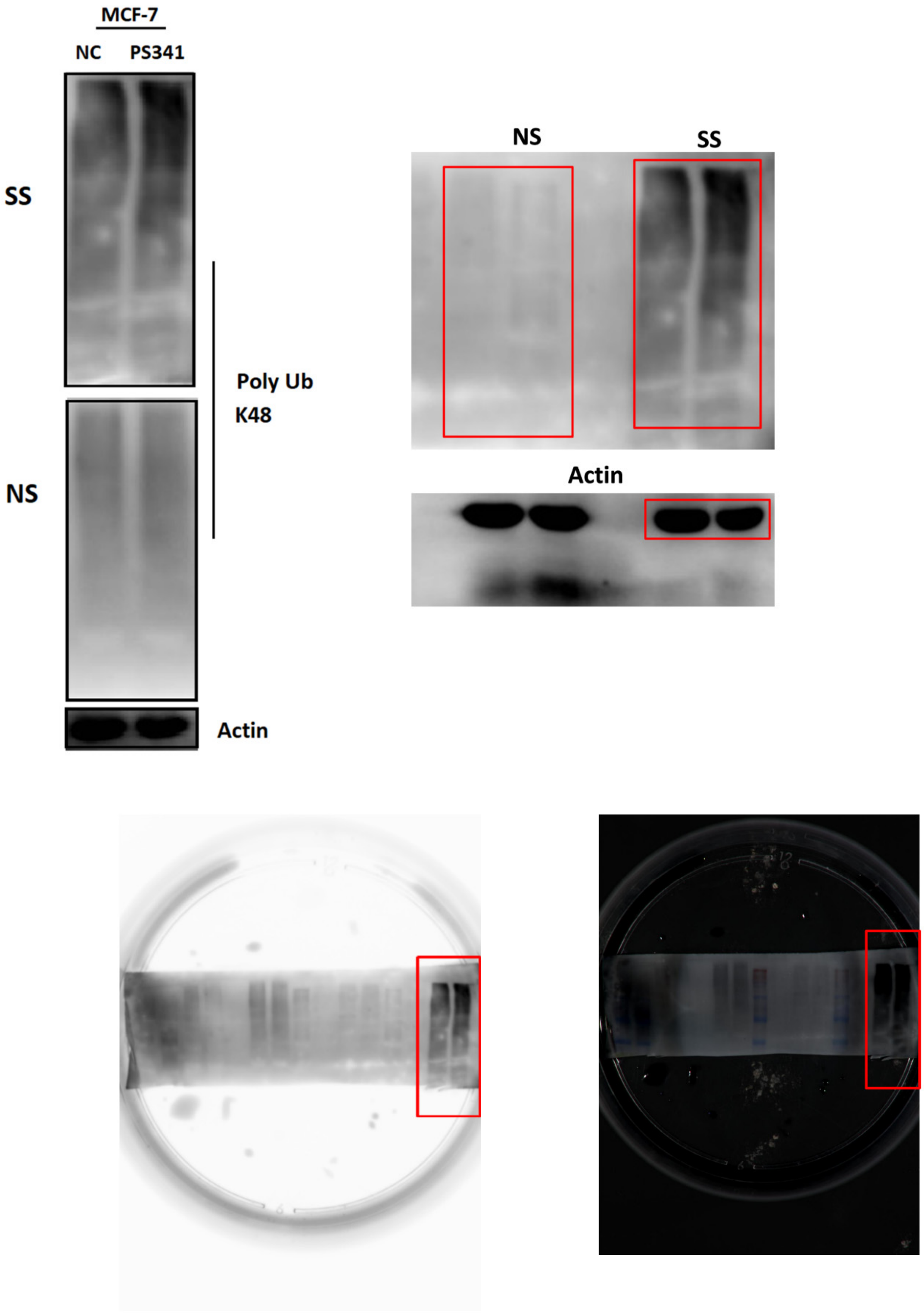

Figure S3. The uncropped western blot images for Figure 3E lysate of MCF-7 treated with PS341, showing Poly Ub K48.

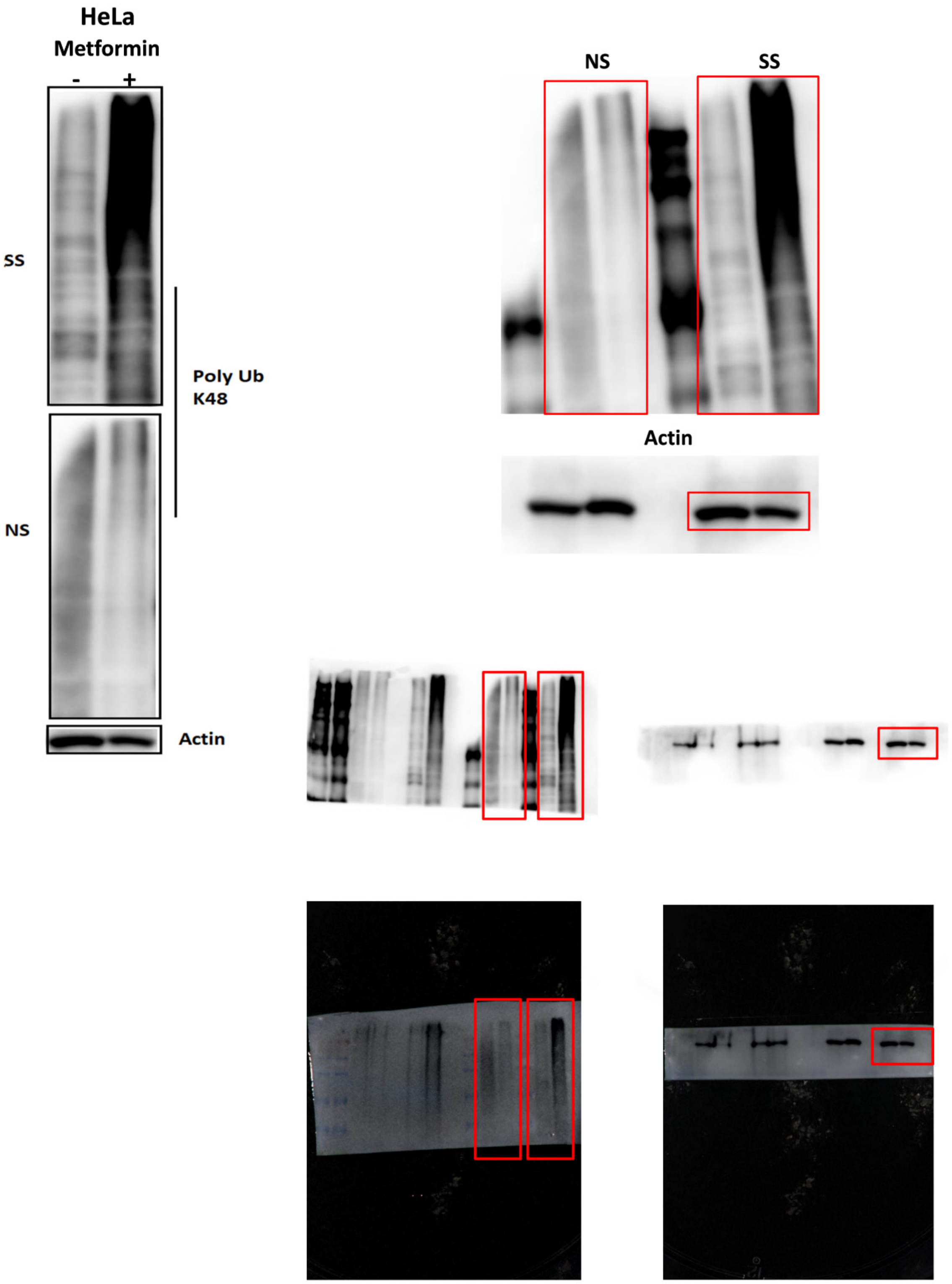

Figure S4. The original western blot images for Figure 3G lysate of HeLa treated with metformin, showing Poly Ub K48 and Actin.

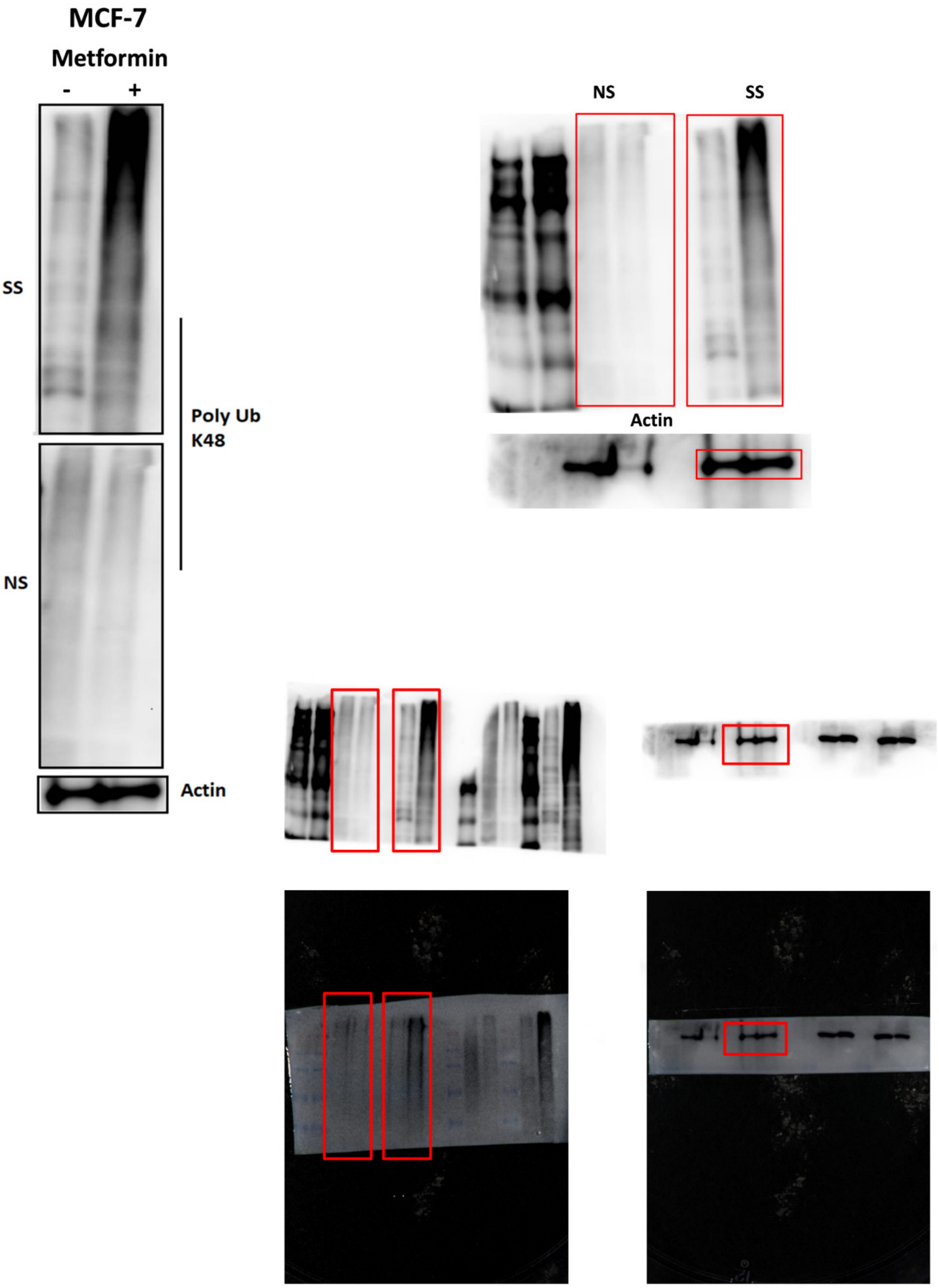

Figure S5. The uncropped western blot images for Figure 3G lysate of MCF-7 treated with metformin, showing Poly Ub K48 and Actin.

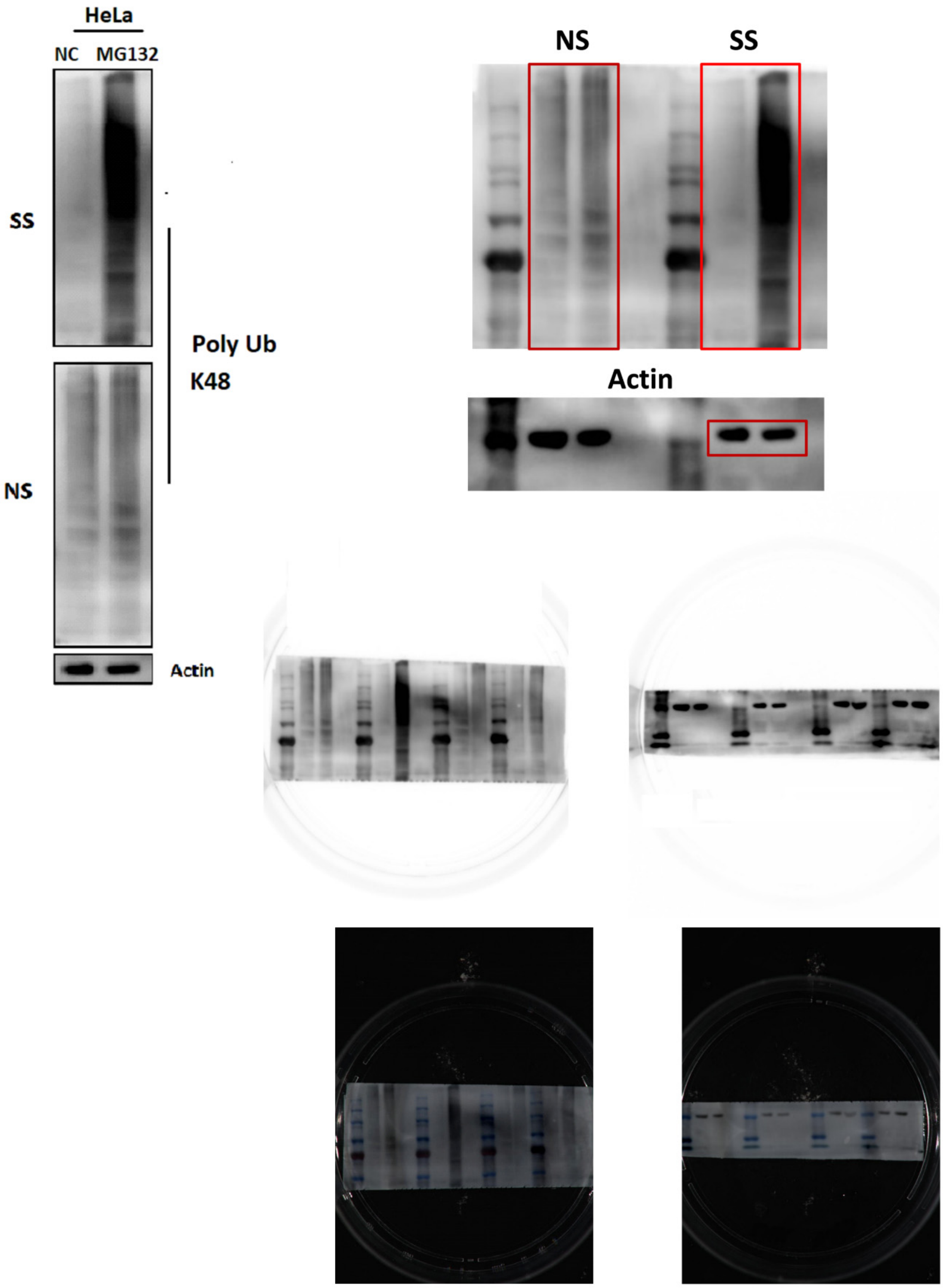

Figure S6. The uncropped western blot images of Suppl Figure 1E lysate of HeLa treated with MG132, showing Poly Ub K48 and Actin.

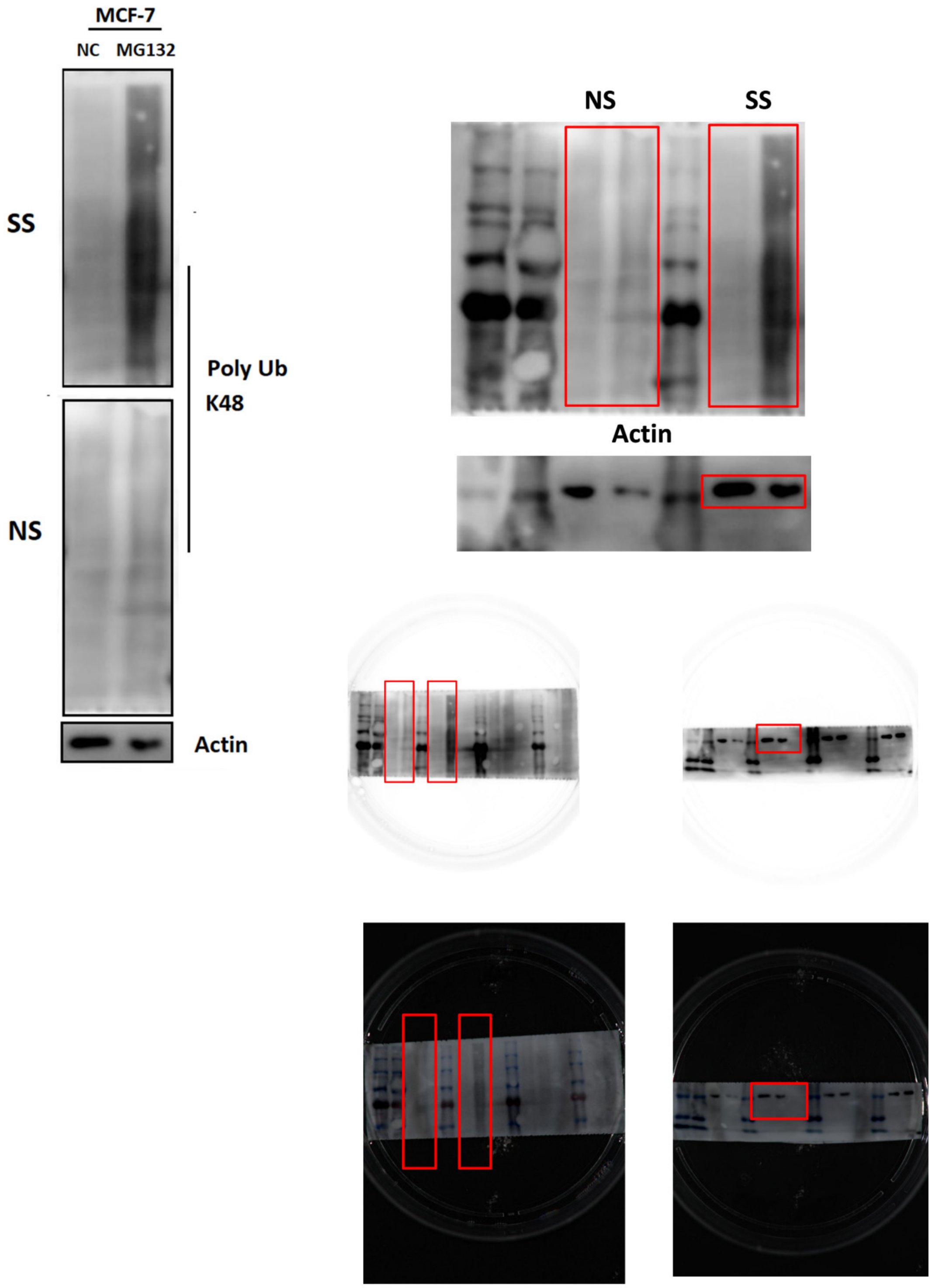

Figure S7. The uncropped western blot images of Suppl Figure 1E lysate of MCF-7 treated with MG132, showing Poly Ub K48 and Actin.

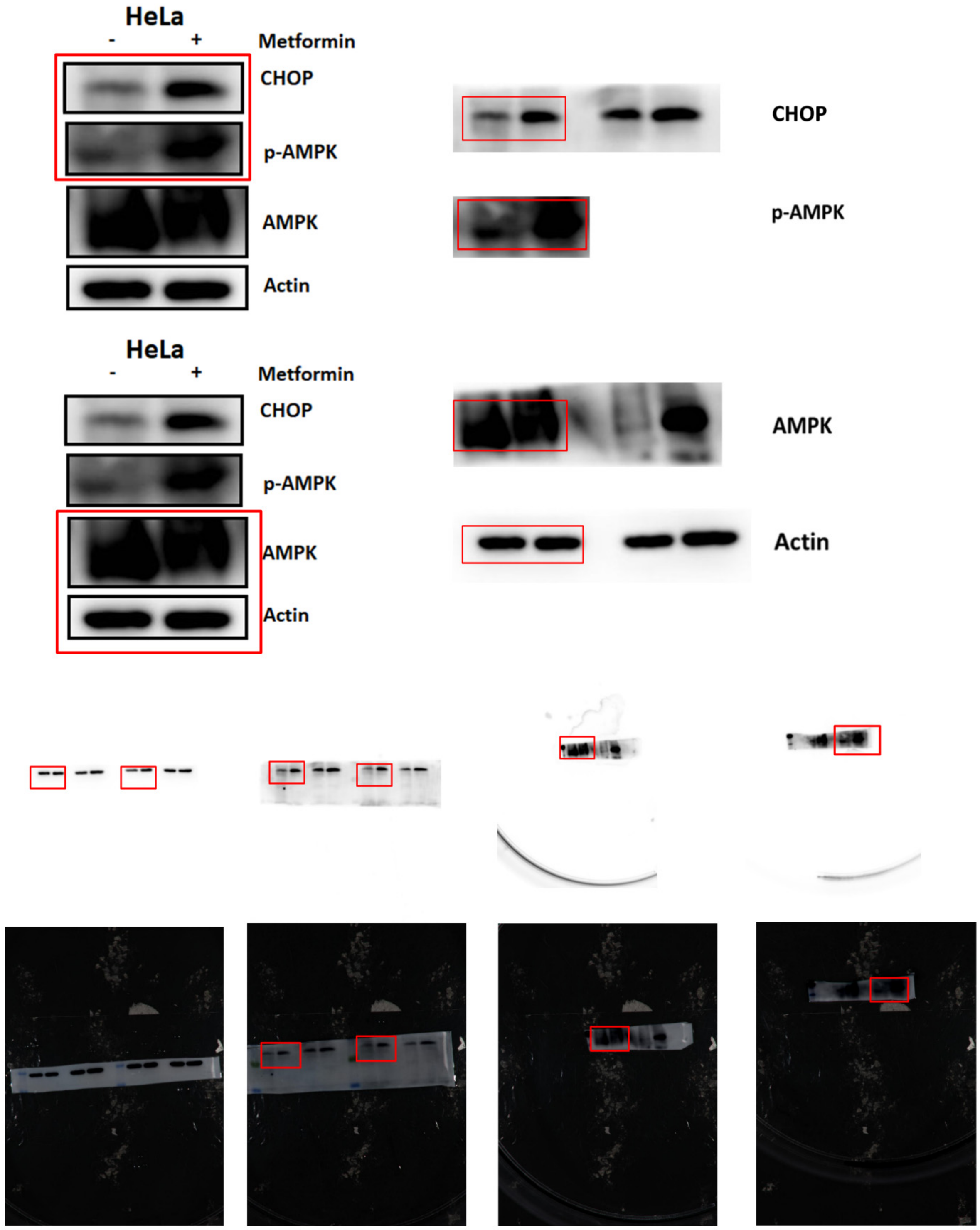

Figure S8. The original western blot images for Figure 4A, lysate of HeLa treated with metformin; CHOP, P-AMPK, AMPK and Actin.

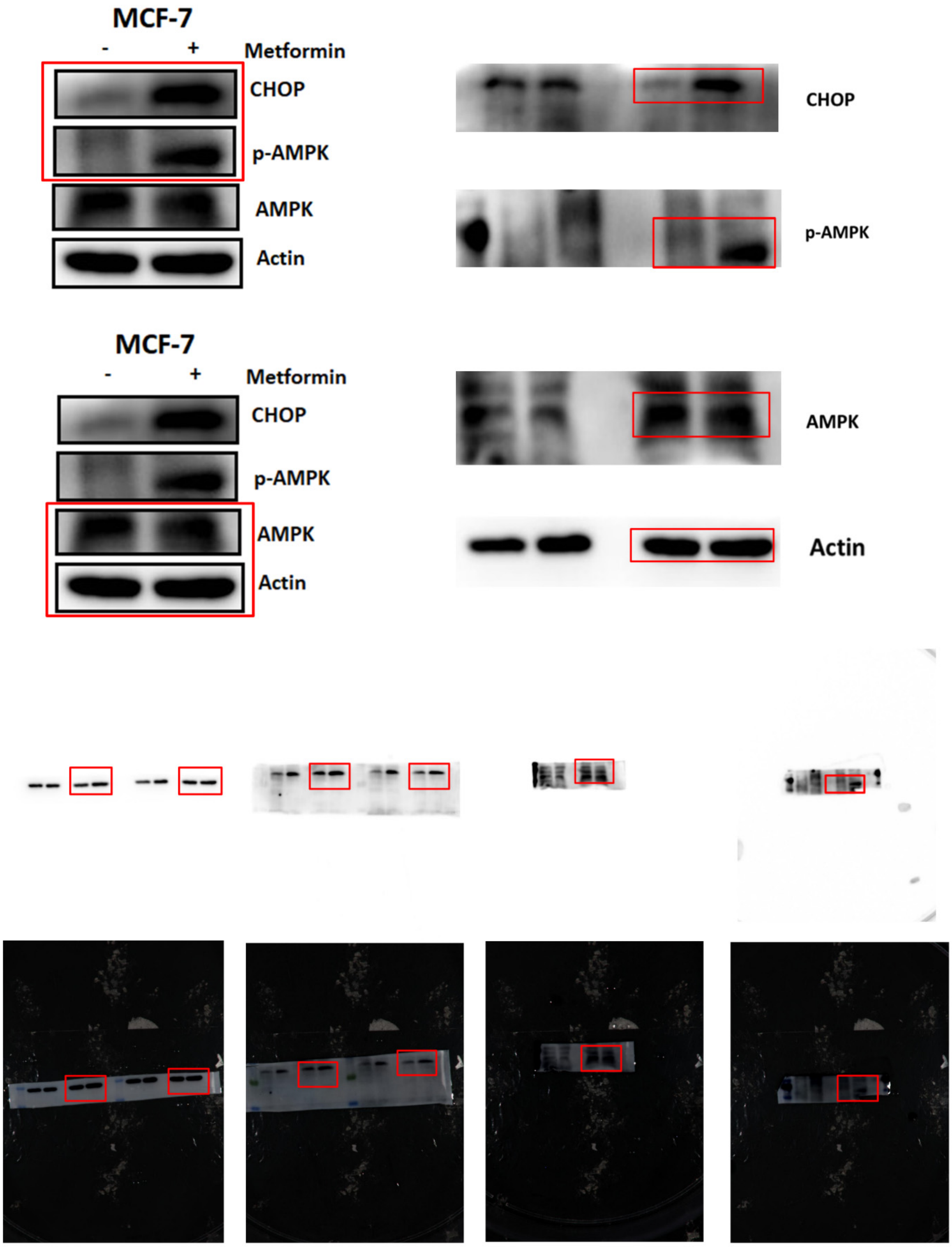

Figure S9. The uncropped western blot images for Figure 4A, lysate of MCF-7 treated with metformin; CHOP, P-AMPK, AMPK and Actin.

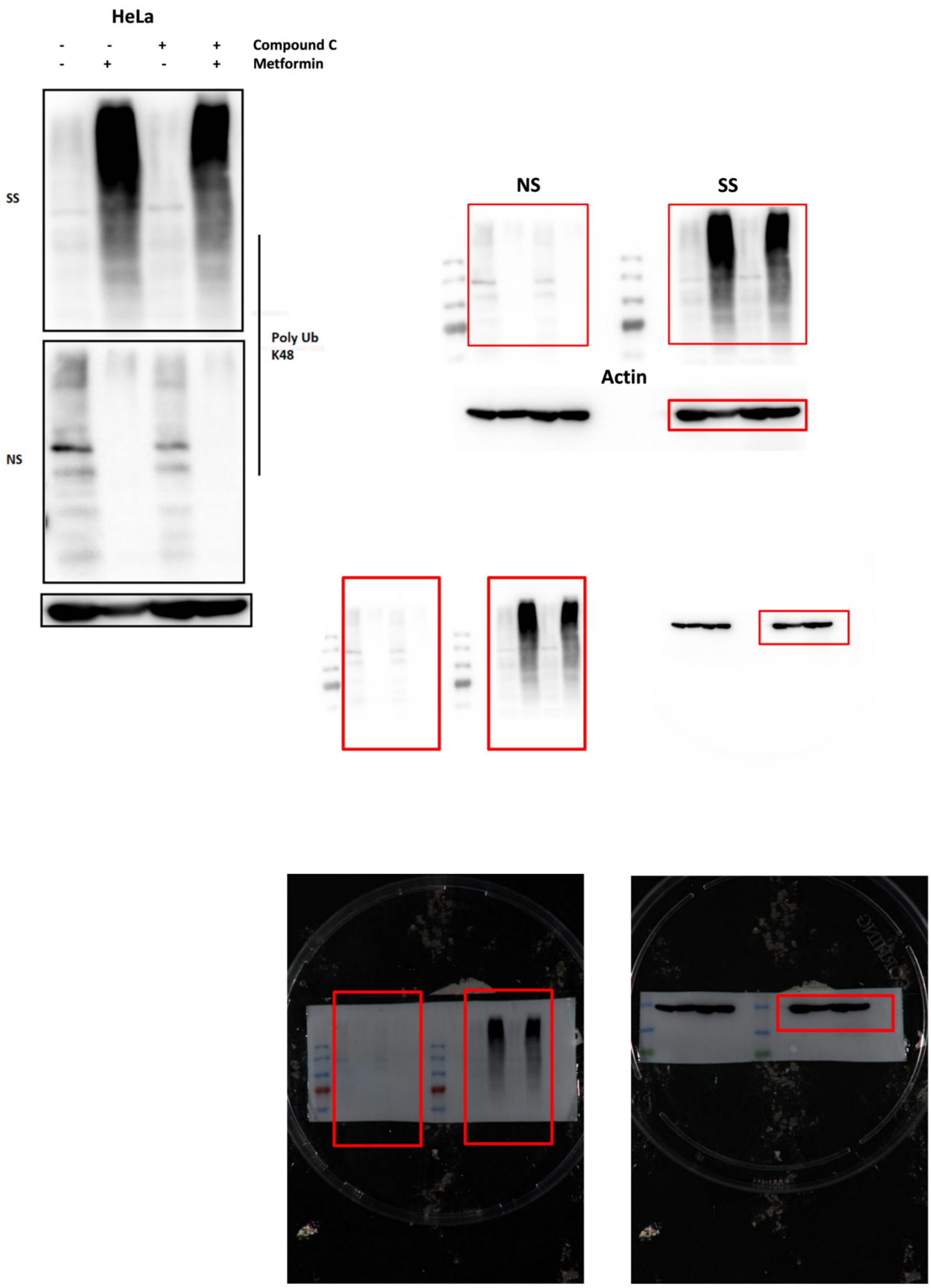

Figure S10. The original western blot images for Figure 4G lysate of HeLa treated with metformin with or without compound C, showing Poly Ub K48 and Actin.

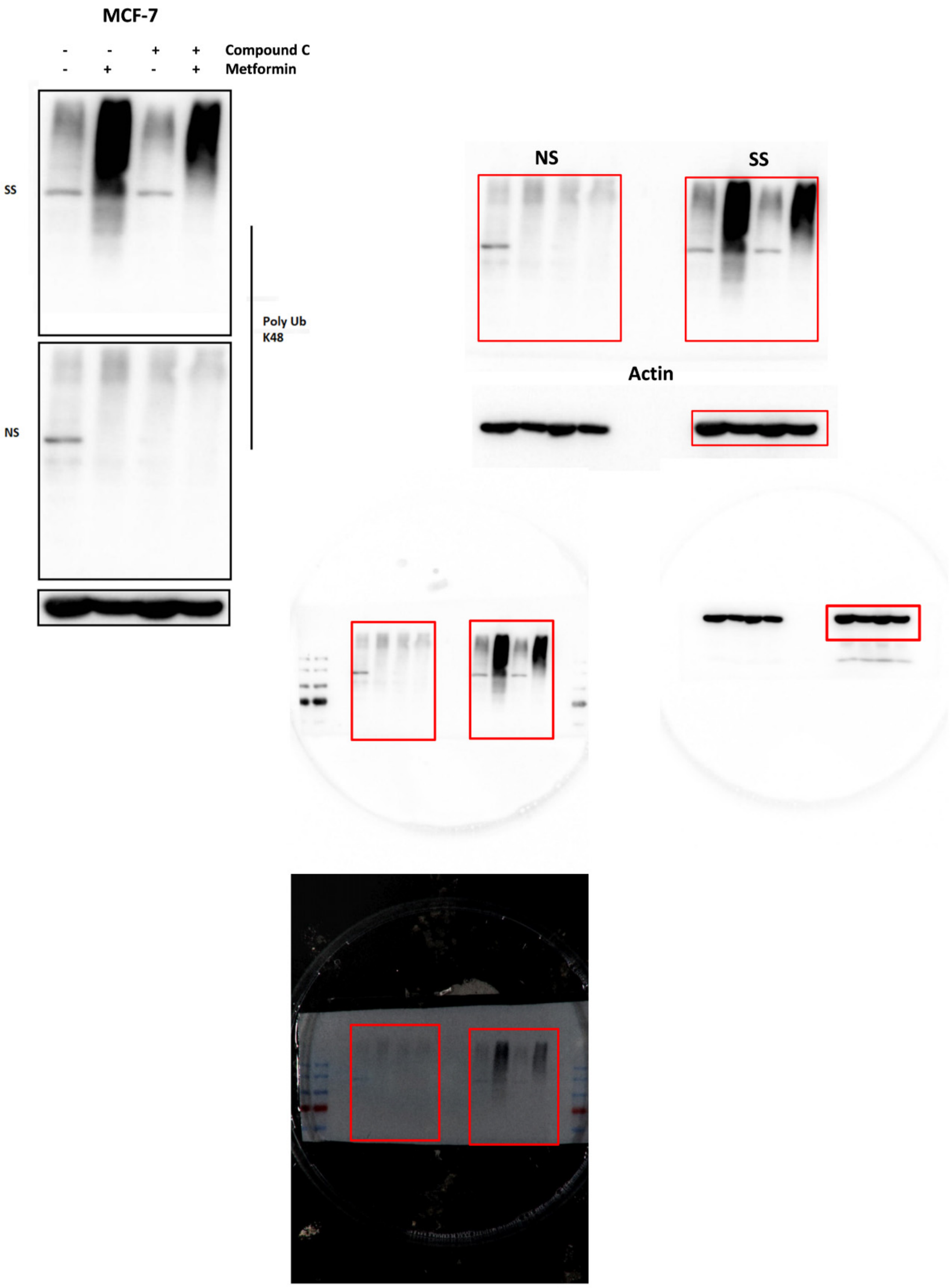

Figure S11. The original western blot images for Figure 4G lysate of MCF-7 treated with metformin with or without compound C, showing Poly Ub K48 and Actin.
